# Prospective Analysis Reveals Associations between Carbohydrate Intakes, Genetic Predictors of Short-Chain Fatty Acid Synthesis, and Colorectal Cancer Risk

**DOI:** 10.1158/0008-5472.CAN-22-3755

**Published:** 2023-04-25

**Authors:** Cody Z. Watling, Rebecca K. Kelly, Neil Murphy, Marc Gunter, Carmen Piernas, Kathryn E. Bradbury, Julie A. Schmidt, Timothy J. Key, Aurora Perez-Cornago

**Affiliations:** 1 Cancer Epidemiology Unit, Nuffield Department of Population Health, University of Oxford, Oxford, United Kingdom.; 2 Nutrition and Metabolism Branch, International Agency for Research on Cancer, Lyon, France.; 3 Nuffield Department of Primary Care, University of Oxford, Oxford, United Kingdom.; 4 National Institute for Health Innovation, School of Population Health, The University of Auckland, Auckland, New Zealand.; 5 Department of Clinical Epidemiology, Department of Clinical Medicine, Aarhus University and Aarhus University Hospital, Aarhus, Denmark.

## Abstract

Whole grain and fiber intakes may decrease the risk of colorectal cancer. The interplay between host genetic factors, colonization of specific bacteria, production of short-chain fatty acids (SCFA), and intake of whole grains and fiber could alter the protective role of carbohydrates against colorectal cancer. Here, we assessed intakes of types and sources of carbohydrates in 114,217 UK Biobank participants with detailed dietary data (2–5 24-hour dietary assessments), and a host polygenic score (PGS) was applied to categorize participants as high or low for intraluminal microbial SCFA production, namely, butyrate and propionate. Multivariable Cox proportional hazards models were used to determine the associations of carbohydrates and SCFA with colorectal cancer incidence. During a median follow-up of 9.4 years, 1,193 participants were diagnosed with colorectal cancer. Risk was inversely associated with intakes of non-free sugar and whole grain fiber. Evidence of heterogeneity was observed by the butyrate PGS; consuming higher amounts of whole grain starch was only associated with a lower risk of colorectal cancer in those with predicted high SCFA production. Similarly, in additional analyses utilizing the larger UK Biobank cohort (*N* = 343,621) with less detailed dietary assessment, only individuals with a high genetically predicted butyrate production had a lower risk of colorectal cancer per 5 g/day intake of bread and cereal fiber. This study suggests that colorectal cancer risk varies by intake of carbohydrate types and sources, and the impact of whole grain intake may be modified by SCFA production.

**Significance:**

Prospective population-level analyses provide evidence supporting the importance of butyrate production in reduction of colorectal cancer risk by whole grain consumption.

## Introduction

Colorectal cancer is the third most common cause of cancer death for both men and women worldwide (1). Smoking, excess adiposity, processed meat intake, and alcohol consumption are well-established modifiable risk factors for colorectal cancer (2, 3). Other dietary factors may also influence colorectal cancer risk; the latest World Cancer Research Fund (WCRF) meta-analysis of prospective studies from 2017 lists foods containing dietary fiber, whole grains, and dairy product consumption as probable factors for decreasing risk and red meat intake as increasing risk (2, 4). However, further research is needed to assess if specific sources and types of carbohydrates relate differently to colorectal cancer risk, as well as to understand potential mechanisms underlying these associations.

One hypothesis that may explain the lower risk of colorectal cancer among individuals who consume more dietary fiber and whole grains is the fermentation of dietary fiber in the colon, which produces the short-chain fatty acids (SCFA) butyrate, propionate, and acetate (5). Evidence from animal and *in vivo* studies has suggested that butyrate, which is used as an energy source by colonocytes, may be protective against carcinogenesis (6–9). Large genetic studies of up to 18,000 individuals have suggested that the composition of microbiota in the gut is influenced by host genetic factors (10, 11). As such, mechanisms may interplay in the colorectum between host genetic factors, colonization of specific bacteria, production of SCFA, and intake of whole grains and fiber in reducing colorectal cancer risk; however, research is needed to understand these potential relationships.

We, therefore, aimed to examine the associations of carbohydrate types and sources with colorectal cancer risk in 114,217 participants from the UK Biobank with detailed dietary data. We also examined if host genetic variants related to colonic/microbial SCFA production modulated the associations of carbohydrate and fiber intakes with the risk of colorectal cancer.

## Patients and Methods

### Study design and participants

In total, 503,317 individuals (5.5% response rate) ages 37 to 73 years provided written informed consent to enroll in the UK Biobank between 2006 and 2010 (12). Eligible people across the UK, living within 30 km of one of the 22 assessment centers (∼9.2 million people), were identified from the National Health Service (NHS) patient registers and invited to participate. At recruitment, participants provided informed consent and detailed information about lifestyle, sociodemographic, and reproductive factors via a self-administered touchscreen questionnaire. Anthropometric measurements were conducted by trained staff using standardized procedures (http://biobank.ctsu.ox.ac.uk/crystal/crystal/docs/Anthropometry.pdf), and blood samples were also collected. Ethical approval was obtained from the North West Multi-Centre Research Ethics Committee (reference number 21/NW/0157) and the UK Biobank is conducted with oversight from the UK Biobank Ethics Advisory Committee. A full description of the study assessment, protocol, and ethical approval can be found on the UK Biobank website (https://www.ukbiobank.ac.uk/).

### Assessment of diet: 24-hour dietary assessment

A subsample of participants completed the Oxford WebQ, a validated 24-hour dietary assessment (13), on at least two occasions (maximum of five; Supplementary Fig. S1). This online assessment asked participants to recall the frequency of consumption of 206 types of foods and 32 types of drinks during the previous 24 hours (14). Further description of the Oxford WebQ can be found in the Supplementary Methods.

### Estimation of nutrient intakes

Carbohydrate intakes were estimated in each 24-hour dietary assessment based on the foods and beverages consumed by participants using the UK Nutrient Databank food composition tables (15–17). Percentages of energy from total carbohydrates, total sugars, free sugars (all monosaccharides and disaccharides added to foods, plus sugars naturally present in honey, syrups and unsweetened fruit juices; ref. 18), non-free sugars (total sugar minus free sugars), total starch, whole grain starch, and refined grain starch were estimated for each 24-hour dietary assessment and were averaged across all available 24-hour dietary assessments for each individual to estimate usual intake of each type and source of carbohydrate. Similarly, total nonstarch polysaccharides (NSP; Englyst fiber; ref. 17), fiber from vegetables, fiber from fruits, and fiber from whole grains were estimated in grams/day (g/day) in each 24-hour dietary assessment and averaged across all available 24-hour dietary assessments for each individual to obtain the average fiber intake from each source. A full description of these calculations can be found in the Supplementary Methods. We also report results for estimated absolute g/day of whole grains and refined grains estimated from the 24-hour dietary assessments for comparison with previous meta-analyses (2).

### Exclusions

Supplementary Fig. S2 presents the flow chart of the exclusions for this analysis. Briefly, participants were excluded if they withdrew their consent (*n* = 922), had a diagnosis of cancer before recruitment (except for nonmelanoma skin cancer; *n* = 29,504), their genetic sex did not match their reported sex (*n* = 321), or they did not complete a 24-hour dietary assessment (*n* = 251,938). We also excluded 24-hour dietary assessments if participants did not report a reliable energy intake [men: >17,575 kJ/d (4,200 kcal) or <3,347 kJ/d (800 kcal); women >14,644 kJ/d (3,500 kcal) or <2,092 kJ/d (500 kcal); ref. 19] or reported that they were ill or fasting on the relevant day (*n* = 2,439 and *n* = 592 participants removed as they no longer had completed a 24-hour dietary assessment, respectively). To reduce random error, participants who did not complete at least two valid 24-hour dietary assessments were excluded (*n* = 100,487). Participants who were censored or diagnosed with cancer before the completion of their final 24-hour dietary assessment were also excluded from this analysis (*n* = 2,897). In total, this left 114,217 participants who completed a minimum of two (maximum of five) valid 24-hour dietary assessments.

### Colorectal cancer incidence

Incident cancer diagnoses were determined using a combination of the NHS Digital records and Public Health England for participants from England and Wales, the NHS Central Register for participants from Scotland as well as Hospital Episode Statistics (HES) data for English participants, and Scottish Morbidity Records (SMR) for Scottish participants (see Supplementary Methods for further detail and UK Biobank website on cancer registry information: https://biobank.ndph.ox.ac.uk/crystal/crystal/docs/CancerLinkage.pdf). Participants contributed follow-up time from date of completion of their last 24-hour dietary assessment until the date of first registration of cancer [excluding nonmelanoma skin cancer (International Classification of Diseases 10th edition (ICD-10): C44)], date of death, or last day of follow-up from HES and SMR data or Welsh cancer registry (September 30, 2021, for English participants, July 31, 2021, for Scottish participants, and February 29, 2020, for Welsh participants). Cancer registry data were available until February 29, 2020, for English participants and January 31, 2021, for Scottish participants. After this time, participants from England and Scotland were followed using HES and SMR databases, respectively; for Wales, hospital episode data were not used because the cancer registry had longer follow-up data. Participants were coded as having an event if they had an incident diagnosis of colorectal cancer (ICD-10 codes: C18 for colon cancer; C19 and C20 for rectal cancer), or if no prior incident diagnosis was reported and their primary underlying cause of death was colorectal cancer.

### Polygenic score

The genotyping of participants in the UK Biobank has been described in detail elsewhere (20). Briefly, the UK Biobank genotyped 488,377 participants using two arrays, namely, the UK BiLEVE Axiom (*n* = 49,950 participants) including 807,411 single-nucleotide polymorphisms (SNP), and the UK Biobank Axiom (*n* = 438,437 participants), which included 825,927 SNPs and shared 95% of marker content with the UK BiLEVE. Over 90 million variants were imputed from this using the Haplotype Reference Consortium and UK10K + 1000 Genomes reference panels (https://biobank.ctsu.ox.ac.uk/crystal/crystal/docs/genotyping_qc.pdf).

To estimate the potential influence of host genetic factors that may affect microbial SCFA production on the associations of intakes of carbohydrate types and sources with colorectal cancer, we used a 9 SNP polygenic score (PGS) for butyrate and a 3 SNP PGS for propionate (21). These SNPs were previously identified as being associated with butyrate and propionate levels in a genome-wide association study of 952 normoglycemic individuals of white European ancestry residents in the Netherlands (21). Further details of calculations of the PGS can be found in Supplementary Methods and Supplementary Table S1. A higher PGS for butyrate and propionate represented a proxy for predicted higher butyrate production in the colon via the PWY-5022 pathway and higher propionate in fecal samples, respectively (21). For genetic analyses, participants were excluded if they did not have genetic information (*n* = 1,998) or reported they were not of white British ancestry (*n* = 16,445). We also excluded participants with low call rates (<98% *n* = 1,326), sex chromosome aneuploidy (*n* = 66), and related individuals (kinship coefficient >0.0884; *n* = 6,965), leaving a maximum of 87,417 participants and 909 cases of colorectal cancer in these analyses.

### Statistical analysis

Participants were categorized into quartiles of percentage of energy intake from each carbohydrate type and source as well as quartiles by g/day of fiber sources. Intakes of carbohydrates were also modeled as a per 5% energy increment for each source, and intakes of fiber sources were modeled as a 5 g/day increment for each fiber source. Baseline characteristics were summarized across quartiles of carbohydrate and fiber intakes.

Cox proportional hazards regressions, with age as the underlying time variable, were used to calculate hazard ratios (HR) and 95% confidence intervals (CI) for colorectal cancer incidence. All covariates added to the model were selected *a priori* based on probable and known risk factors for colorectal cancer. Minimally adjusted models were stratified by sex and age at recruitment and adjusted for region at recruitment.

The main multivariable Cox regression models were further adjusted for height, physical activity, Townsend deprivation index, education, employment status, smoking status, alcohol consumption estimated from the recruitment questionnaire, ethnicity, diabetes status, nonsteroidal anti-inflammatory drug (NSAID) use, body mass index (BMI), consumption of red and processed meat from the touchscreen questionnaire at recruitment, intake of fruit and vegetables estimated from the 24-hour dietary assessments (excluded from the model when the exposures were total fiber, fiber from fruit and/or vegetables and non-free sugars), energy intake, and women-specific covariates: menopausal hormone therapy use and menopausal status. For covariates in which responses were unknown or missing, participants were categorized into an unknown/missing category. Further explanation of the categorization of covariates can be found in the Supplementary Methods.

The likelihood ratio test (LRT) χ^2^ statistic for including the carbohydrate types or sources or fiber source was estimated using LRTs comparing models without the carbohydrate/fiber source to models with the carbohydrate/fiber source (modeled as 5% energy increment for carbohydrates and 5 g/day for fiber). The χ^2^ value provides a quantitative measure of the extent intake of carbohydrate types or sources improves model fit for colorectal cancer risk. To assess for residual confounding and the extent to which adjustment for confounders influenced the associations of intakes of carbohydrate types and sources with risk of colorectal cancer, the change of χ^2^ values was estimated using minimally adjusted models χ^2^ as the reference value (22, 23). Further details on this method to assess for residual confounding are provided in the Supplementary Methods. LRT for departures from linearity was also estimated by comparing models with carbohydrates (per 5% energy intake/day) to models with carbohydrate quartiles and models with fiber as a continuous variable (per 5 g/day) with models as fiber quartiles, and no evidence of nonlinearity was observed (data not presented).

### Subgroup and sensitivity analyses

We assessed whether there was heterogeneity by sex or by BMI (∼ median: <27, and ≥27 kg/m^2^) by using LRTs comparing the main model to a model including an interaction term between the carbohydrate source (5% energy increment) or fiber (5 g/day increment) and sex or BMI. We further explored if associations varied by tumor site (colon or rectal). In 68 instances, the diagnosis of colon and rectal cancer coincided, and these participants were removed from the subgroup analyses by tumor site. For heterogeneity by tumor site, we stratified Cox models using a competing risks approach (24) and compared the risk coefficients and standard errors of each carbohydrate source as a 5% energy increment, or 5 g/day for fiber, using colon cancer and rectal cancer as separate outcomes.

To assess the robustness of results, sensitivity analyses were conducted restricting to participants who completed a minimum of three 24-hour dietary assessments to further reduce random measurement error in the carbohydrate and fiber intake estimates. To investigate the potential for reverse causality, we removed cases diagnosed in the first 2 years of follow-up and participants who were censored in the first 2 years.

### Genetic factors

To assess for an effect modification between host genetically predicted SCFA production PGS and intakes of carbohydrate types and sources and colorectal cancer risk, we used an LRT comparing the main multivariable model with a model with an interaction term between the carbohydrate source (per 5% energy increment) or fiber intake (per 5 g/day increment) with the butyrate PGS or propionate PGS, separating participants by the median PGS for both butyrate and propionate (low or high PGS). We also adjusted models for the first 10 principal components of ancestry to reduce the potential impact of population stratification.

We further conducted a sensitivity analysis assessing intakes of fiber from breads and cereals derived from the few dietary questions asked at the baseline questionnaire (25) with colorectal cancer risk by the butyrate and propionate PGS in the larger UK Biobank sample (*n* = 343,621; 4,191 cases of colorectal cancer). The fiber from breads and cereals variable was calculated using the consumption frequency and type of bread and cereals reported by individuals at their recruitment visit. Specifically, participants were asked how many slices of bread or bowls of cereal they consumed per week as well as the type of bread and cereal they mainly consumed. From these responses, the NSP contents (in grams) of slices of bread and breakfast cereals were determined based on the frequency of consumption and types of bread or cereal reported in the touchscreen questionnaire. The estimated intakes of fiber from cereal and fiber from bread were then summed to obtain the total intake of fiber from breads and cereals. Further details of the calculation of the fiber from breads and cereals from the touchscreen questionnaire have been described in greater detail elsewhere (25) and further details of this sensitivity analysis can be found in the Supplementary Methods.

All analyses were conducted using Stata version 17.0, and *P* values were two-sided with *P* <0.05 being considered statistically significant.

### Data availability statement

The data analyzed in this study were obtained from the UK Biobank under application 24494. Bona fide researchers can apply to use the UK Biobank data set by registering and applying at http://ukbiobank.ac.uk/register-apply/. All other raw data are available upon request from the corresponding author.

## Results

Over a median of 9.4 years of follow-up, 1,193 incident cases of colorectal cancer occurred. Table 1 presents participants’ characteristics between those in the lowest and highest quartiles of whole grains, refined grains, and total fiber intake and Supplementary Tables S2 and S3 present the baseline characteristics by quartiles for total carbohydrates, sugars, and fiber sources. Supplementary Fig. S3 presents the top food group contributors for each carbohydrate type and source. Participants in the highest quartile of fiber intake had a lower BMI, reported a lower intake of processed and red meat, consumed less alcohol, reported a higher energy intake, and were more likely to be never smokers in comparison with the lowest quartile of fiber intake (Table 1). Participants who consumed the highest amount of whole grain starch were more likely to have a lower BMI, be never smokers, consume less red and processed meat, and have a university or college degree in comparison with those in the lowest quartile (Table 1), and similar differences in characteristics were observed for participants in the highest intake of fiber from whole grains (Supplementary Table S3).

**Table 1.** Baseline characteristics by lowest and highest quartile of intake of whole grain starch, refined grain starch, and fiber in 114,217 UK Biobank participants.

	Whole grain starch		Refined grain starch		Total fiber	
	Q1	Q4	Q1	Q4	Q1	Q4
No. of participants, *N*	28,555	28,554	28,555	28,554	28,555	28,554
Intake of carbohydrate of interest^a^	0.6 (0.8)	10.9 (3.1)	4.4 (2.0)	19.9 (3.9)	11.3 (2.1)	25.5 (4.2)
Sex, male, *N* (%)	13,296 (46.6%)	13,392 (46.9%)	12,402 (43.4%)	13,664 (47.9%)	12,208 (42.8%)	14,356 (50.3%)
Age at recruitment, years	55.0 (7.9)	56.6 (7.6)	57.6 (7.3)	53.8 (8.0)	54.8 (7.8)	56.7 (7.8)
Body mass index, kg/m^2^	27.4 (4.8)	26.0 (4.3)	26.5 (4.5)	27.0 (4.8)	27.2 (4.7)	26.2 (4.5)
Height, cm	169.4 (9.2)	169.7 (9.2)	169.2 (9.1)	169.7 (9.2)	168.4 (9.1)	170.8 (9.3)
Physical activity, high, *N* (%)	4,734 (16.6%)	5,110 (17.9%)	5,475 (19.2%)	4,343 (15.2%)	4,154 (14.5%)	6,199 (21.7%)
Townsend deprivation index, *N* (%)						
Q1. Most affluent	5,757 (20.2%)	6,398 (22.4%)	6,320 (22.1%)	5,891 (20.6%)	5,913 (20.7%)	6,172 (21.6%)
Q5. Most deprived	5,325 (18.6%)	4,446 (15.6%)	4,317 (15.1%)	5,184 (18.2%)	5,235 (18.3%)	4,399 (15.4%)
In paid employment, *N* (%)	18,669 (65.4%)	17,061 (59.7%)	16,134 (56.5%)	19,652 (68.8%)	18,968 (66.4%)	16,717 (58.5%)
University/college degree, *N* (%)	20,111 (70.4%)	21,618 (75.7%)	21,018 (73.6%)	20,645 (72.3%)	19,570 (68.5%)	21,962 (76.9%)
Ethnicity, *N* (%)						
White	27,323 (95.7%)	27,708 (97.0%)	27,908 (97.7%)	26,945 (94.4%)	27,264 (95.5%)	27,650 (96.8%)
Mixed race/Other	404 (1.4%)	264 (0.9%)	227 (0.8%)	499 (1.7%)	361 (1.3%)	322 (1.1%)
Asian or British Asian	426 (1.5%)	300 (1.1%)	159 (0.6%)	718 (2.5%)	472 (1.7%)	302 (1.1%)
Black or Black British	288 (1.0%)	207 (0.7%)	180 (0.6%)	299 (1.0%)	363 (1.3%)	172 (0.6%)
Never smoker, *N* (%)	14,993 (52.5%)	17,547 (61.5%)	16,034 (56.2%)	16,907 (59.2%)	15,322 (53.7%)	17,025 (59.6%)
Diabetes, yes, *N* (%)	1,088 (3.8%)	1,150 (4.0%)	1,000 (3.5%)	1,223 (4.3%)	1,033 (3.6%)	1,167 (4.1%)
NSAID, regular user, *N* (%)	7,585 (26.6%)	6,650 (23.3%)	7,074 (24.8%)	7,291 (25.5%)	7,604 (26.6%)	6,843 (24.0%)
Postmenopausal at recruitment, *N* (%)	9,626 (63.1%)	10,747 (70.9%)	12,370 (76.6%)	8,329 (56.0%)	10,312 (63.1%)	10,206 (71.9%)
Menopausal hormone therapy use, current use, *N*(%)	1,320 (8.7%)	1,090 (7.2%)	1,395 (8.6%)	1,096 (7.4%)	1,518 (9.3%)	1,050 (7.4%)
**Diet variables**						
Alcohol intake, g/day	19.7 (20.9)	13.5 (13.5)	17.4 (17.6)	14.7 (16.1)	19.0 (19.9)	14.4 (15.3)
Red and processed meat intake, 4+ times/wk, *N* (%)	13,034 (45.6%)	9,070 (31.8%)	9,918 (34.7%)	11,818 (41.4%)	11,914 (41.7%)	9,546 (33.4%)
Vegetable and fruit intake, g/day	342.0 (226.6)	409.4 (219.0)	460.8 (250.5)	307.5 (193.1)	219.7 (126.1)	578.5 (256.7)
Total carbohydrate intake, % energy	46.6 (8.1)	52.1 (6.5)	47.6 (8.4)	51.5 (6.5)	46.2 (8.2)	52.2 (6.5)
Total sugar intake, % energy	23.0 (7.0)	25.2 (6.1)	26.3 (7.1)	22.1 (5.9)	22.7 (6.8)	26.2 (6.4)
Total starch intake, % energy	23.6 (6.3)	26.8 (5.1)	21.3 (5.6)	29.3 (4.6)	23.4 (6.2)	25.9 (5.4)
Total fiber intake, g/day	14.9 (5.1)	20.5 (5.7)	19.2 (6.2)	16.3 (5.1)	11.3 (2.1)	25.4 (4.1)
Total fiber from whole grains, g/day	0.5 (0.8)	7.2 (2.8)	4.8 (3.4)	2.4 (2.4)	1.7 (1.7)	5.9 (3.6)
Total energy intake, kJ/day	8,696 (2,067)	8,257 (1,831)	8,246 (1,886)	8,720 (1,999)	7,393 (1,617)	9,845 (1,913)

Note: Values are mean (SD) unless otherwise indicated.

Abbreviations: g, grams; g/day, grams per day; kJ, kilojoules; *N*, number of participants; NSAID, nonsteroidal anti-inflammatory drug; Q, quantile; wk, week; y, years.

^a^Values represent percentage of energy for whole grain starch and refined grain starch and g/day for total fiber.

**Figure 1.**
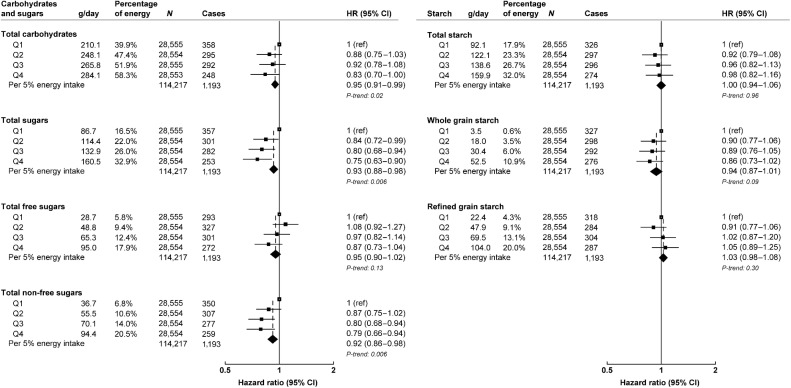
Multivariable-adjusted hazard ratios (95% CI) for colorectal cancer risk by the percentage of energy intake from carbohydrates, sugars, and starches. All models are stratified by sex, age at recruitment, and adjusted for region of recruitment, body mass index, height, physical activity, Townsend deprivation index, education, smoking, alcohol consumption, ethnicity, diabetes status, nonsteroidal anti-inflammatory drug use, red and processed meat intake, fruit and vegetables intake (except for non-free sugars), energy intake, and female-specific covariates: menopausal hormone therapy use, and menopausal status. g/day and percentage of energy from carbohydrate, sugars, and starch calculated as mean per day within each quartile. g/day, grams per day; *N*, number of participants; Q, quartile; ref, reference group.

Multivariable-adjusted HRs for intakes of total carbohydrates and carbohydrates from different types and sources and colorectal cancer risk are shown in Fig. 1 (see Supplementary Table S4 for minimally adjusted results and sequential adjustments of potential confounders). Intake of total carbohydrates was associated with a lower risk of colorectal cancer (HR per 5% energy increment: 0.95; 95% CI, 0.91–0.99; Fig. 1); however, the χ^2^ value was reduced by >70% in comparison with minimally adjusted models (Supplementary Table S4). For every 5% higher energy intakes of total sugars and non-free sugars, an inverse association with colorectal cancer was observed (HR: 0.93, 0.88–0.98 and 0.92, 0.86–0.98, respectively; Fig. 1). No statistically significant association was observed for starch from whole grain and colorectal cancer risk (HR per 5% energy increase: 0.94, 0.87–1.01, *P-*trend *=* 0.09); however, when whole grain intake was modeled as absolute grams of whole grain foods instead of % energy from starch from whole grain, an inverse association with colorectal cancer risk was observed (HR per 50 g/day intake:0.95, 0.91–0.98, *P* = 0.004; Supplementary Table S5). No other associations between carbohydrate intake and colorectal cancer risk were observed.

**Figure 2.**
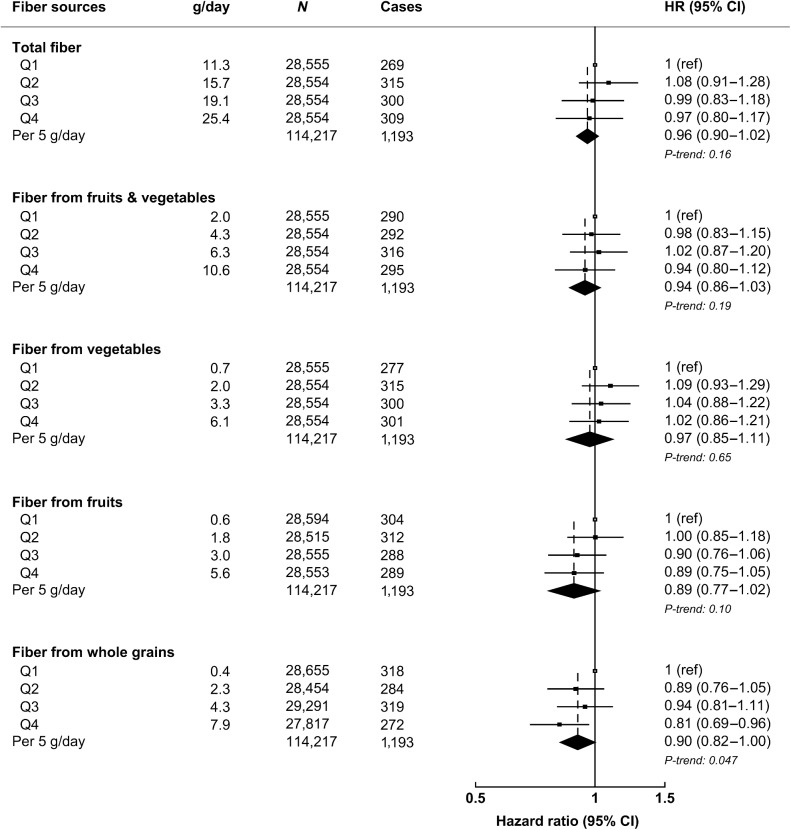
Multivariable-adjusted hazard ratios (95% CI) for colorectal cancer risk by total fiber and fiber from various sources. All models are stratified by sex, age at recruitment, adjusted for region of recruitment, body mass index, height, physical activity, Townsend deprivation index, education, smoking, alcohol consumption, ethnicity, diabetes status, nonsteroidal anti-inflammatory drug use, red and processed meat intake, fruit and vegetable intake (except for total fiber, and fiber from fruits and/or vegetables), energy intake, and female-specific covariates: menopausal hormone therapy use and menopausal status. g/day of fiber calculated as mean intake per day within each quartile. g/day, grams per day; *N*, number of participants Q, quartile; ref, reference group.

Multivariable-adjusted HRs for fiber intake and colorectal cancer are presented in Fig. 2 (see Supplementary Table S6 for minimally adjusted results and sequential adjustments of potential confounders). No associations were observed for total fiber intake; however, a borderline inverse association for fiber from whole grains with colorectal cancer risk was observed (HR per 5 g/day increment in intake: 0.90, 0.82–1.00, *P* = 0.047; Fig. 2) and participants in the highest quartile of intake had a lower risk of colorectal cancer in comparison with those in the lowest quartile of intake (HR_Q4 vs. Q1_: 0.81, 0.69–0.96).

### SCFA polygenic scores

**Figure 3.**
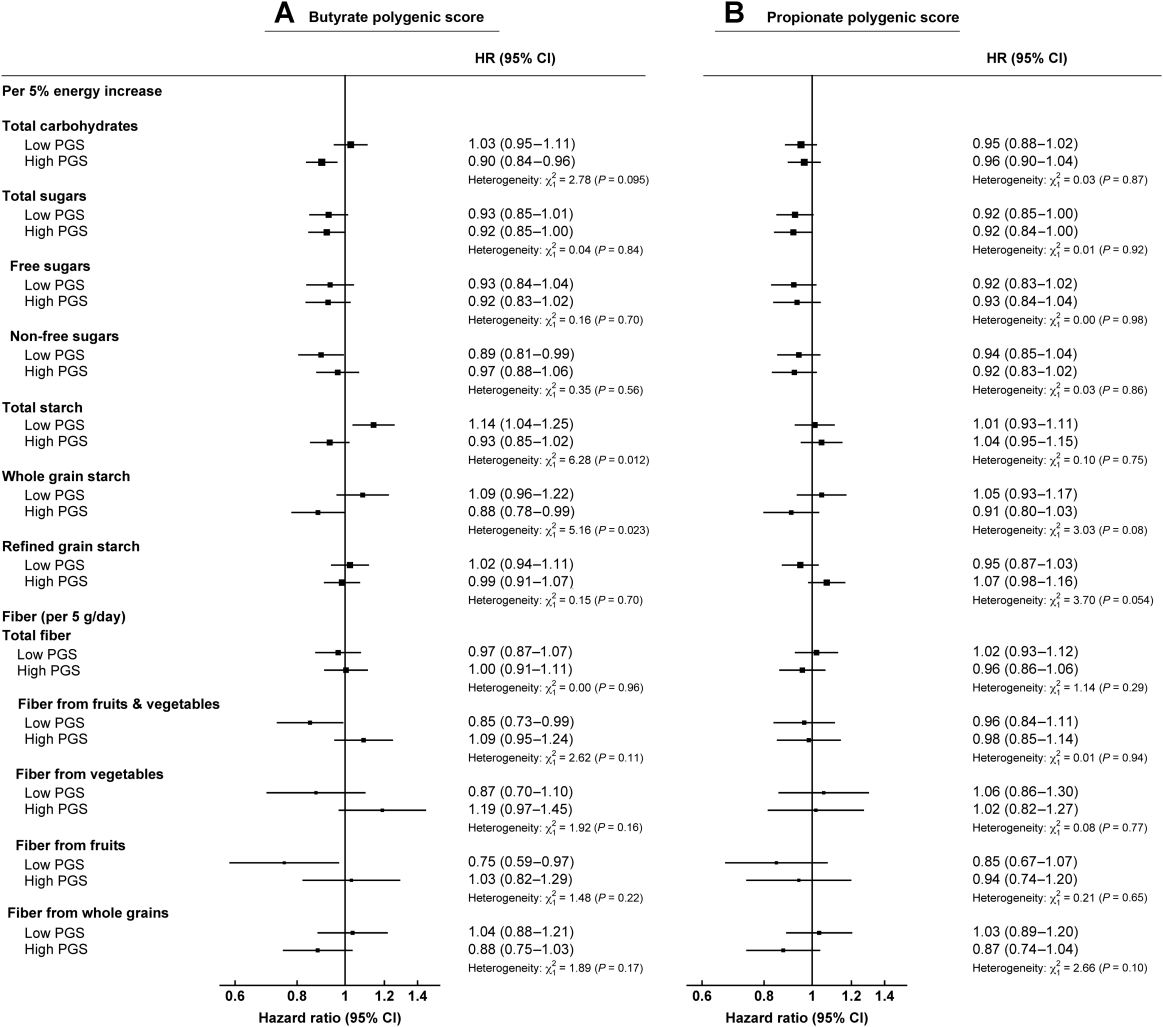
Multivariable-adjusted hazard ratios (95% CI) for intake of carbohydrates and fiber and colorectal cancer risk separated by genetically predicted host short-chain fatty acid production for butyrate (**A**) and propionate (*n* = 87,417; **B**). All models are stratified by sex, age at recruitment, adjusted for region of recruitment, first 10 genetic principal components, body mass index, height, physical activity, Townsend deprivation index, education, smoking, alcohol consumption, diabetes status, nonsteroidal anti-inflammatory drug use, red and processed meat intake, fruit and vegetable intake (except when fiber from vegetables and/or fruits, and non-free sugar intake was the exposure), energy intake, and female-specific covariates: menopausal hormone therapy use and menopausal status. Analyses are restricted to white British participants. χ^2^ and *P* value represents improvement of fit obtained from likelihood ratio tests for including an interaction term between butyrate or propionate polygenic score and carbohydrate type/source (modeled as a 5% energy increment) or fiber source (modeled as a 5 gram/day increment) into the model.

In participants with a high PGS for butyrate production, intake of whole grain starch was inversely associated with colorectal cancer risk (HR per 5% energy: 0.88, 0.78–0.99), whereas no association was observed between intake of starch from whole grain and colorectal cancer risk for individuals with a low host butyrate PGS (HR: 1.09, 0.96–1.22; *P*_heterogeneity_ = 0.023; Fig. 3A). Participants consuming higher amounts of total starch and being in the low butyrate PGS category had a higher risk of colorectal cancer (HR per 5% energy: 1.14, 1.04–1.25), whereas no association was observed between total starch intake and risk among individuals with a high butyrate PGS (HR: 0.93, 0.85–1.02; *P*_heterogeneity_ = 0.012; Fig. 3A). No evidence of heterogeneity was observed for intakes of carbohydrate types and sources and colorectal cancer risk by propionate PGS (Fig. 3B).

#### Replication in a larger UK biobank sample

**Figure 4.**
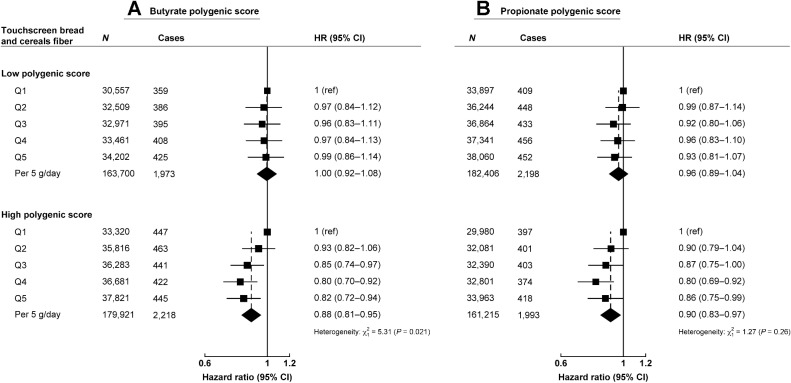
Multivariable-adjusted hazard ratios (95% CI) for intake of fiber from breads and cereals from the touchscreen questionnaire and colorectal cancer risk by genetically predicted host short-chain fatty acid production for butyrate (**A**) and propionate (*n* = 343,621; **B**). Models stratified for sex and age at recruitment, and further adjusted for region, first 10 principal components, height, physical activity, Townsend deprivation index, education, employment, smoking, alcohol consumption measured at recruitment, diabetes status, nonsteroidal anti-inflammatory drug use, body mass index, processed and red meat intake, and female-specific covariates: menopausal hormone therapy use and menopausal status. Analyses are restricted to white British participants. χ^2^ and *P* value represents improvement of fit obtained from likelihood ratio tests for including an interaction term between butyrate or propionate polygenic score and fiber from breads and cereals (modeled as a 5 gram/day increment) into the model. g/day, grams per day; *N*, number of participants; Q, quintile; ref, reference group.

In additional analyses using the intake of fiber from breads and cereals calculated from dietary questions asked in the touchscreen questionnaire completed by the larger UK Biobank sample (*n* = 343,621), we observed evidence of heterogeneity by the butyrate production PGS: a 5 g/day higher intake of fiber from breads and cereals was inversely associated with colorectal cancer risk for individuals with a high butyrate PGS (HR per 5 g/day increase:0.88, 0.81–0.95), whereas no association was observed for individuals in the low butyrate PGS (HR: 1.00, 0.92–1.08; *P*_heterogeneity_ = 0.021; Fig. 4A). Similar associations were also observed for the propionate production PGS; however, the test for heterogeneity was nonsignificant (*P*_heterogeneity_ = 0.26; Fig. 4B).

### Subgroup analyses

No evidence of heterogeneity across sex and BMI categories was observed for the main analyses using 24-hour dietary assessments (Supplementary Figs. S4 and S5). Some evidence of heterogeneity was observed when looking at tumor sites (i.e., colon and rectal); non-free sugar intake was inversely associated with rectal cancer risk (HR: 0.81, 0.73–0.90) but was not associated with colon cancer risk (HR: 0.98, 0.91–1.05; *P*_heterogeneity_ = 0.003; Supplementary Fig. S6). Similarly, evidence of heterogeneity was observed for intake of fiber from fruit, which was inversely associated with rectal cancer risk (HR: 0.62, 0.47–0.83) but was not associated with colon cancer risk (HR: 1.04, 0.87–1.25; *P*_heterogeneity_ = 0.003; Supplementary Fig. S6).

### Sensitivity analyses

In sensitivity analyses restricting to participants who completed a minimum of three 24-hour dietary assessments (total *N* = 69,223 and cases = 734) and removing the first 2 years of follow-up (total *N* = 111,724; and cases = 978), associations remained largely unchanged with CIs widening, probably due to a smaller number of cases and participants, and thus reduced statistical power (Supplementary Table S7; Supplementary Table S8).

## Discussion

In this analysis of 114,217 participants from the UK Biobank, intakes of carbohydrates, total sugar, non-free sugars, total whole grains, and fiber from whole grains were inversely associated with colorectal cancer risk. Moreover, we found suggestive evidence of effect modification by host genetically predicted SCFA production, with higher intakes of starch from whole grains being inversely associated with colorectal cancer risk only in those with a high host genetically predicted butyrate. Similar evidence of heterogeneity was observed for intake of fiber from cereals and breads in the larger UK Biobank sample (*n* = 343,621) with less detailed dietary questions.

In this study, higher intakes of total carbohydrates were inversely associated with colorectal cancer risk; however, the χ^2^ value was reduced significantly when the minimally adjusted model was adjusted for additional confounders, which suggests residual confounding may operate (22). Moreover, intakes of total sugars and non-free sugars were inversely associated with colorectal cancer risk. The majority of intake of total sugars in this cohort was from non-free sugar sources, and this is likely why an inverse association was also observed for total sugar intake. Participants consuming a higher percentage of energy from non-free sugars in this cohort consumed greater amounts of fruits and vegetables, for which there is limited evidence for a possible protective association with colorectal cancer (2). The association of non-free sugars with colorectal cancer may also be partly due to intake of dairy products, a source of non-free sugars (∼23% of all non-free sugars) in this population (26). Dairy product consumption has been suggested by the WCRF as a probable factor for reducing the risk of colorectal cancer (2, 27), potentially due to the high calcium content (28). In contrast, there was no association between free sugar intake and colorectal cancer risk, which is similar to previous findings from prospective cohort studies looking at intakes of free sugar sources such as fructose and sugar-sweetened beverages (29, 30). We observed evidence of heterogeneity by tumor site with an inverse association of non-free sugars with rectal cancer, but not with colon cancer. Existing evidence does not suggest differences in colorectal cancer risk by tumor sites for dietary factors such as fiber, whole grains, and processed meat (2), and mechanisms for any potential difference are not clear; therefore, these results may be due to chance. However, potential differences in risk could be attributable to microbial and physiologic variation through the large intestine (31), but whether this influences risk for dietary factors remains unclear.

Higher intakes of carbohydrate sources of low nutritional quality, such as refined grains and free sugars, may be associated with higher risks of hyperinsulinemia (32) and obesity (33), which are risk factors for colorectal cancer (2, 34). However, in analyses adjusted and not adjusted for BMI we did not observe any associations of refined grain starch or free sugar intake with colorectal cancer risk, which is in line with previous studies (4, 30, 35, 36).

In the latest WCRF meta-analysis from 2017, a higher intake of dietary fiber was associated with a 7% lower risk of colorectal cancer per 10 g/day increment (2). Total fiber intake was not associated with colorectal cancer risk in our main analyses, although we did observe an inverse association for fiber from whole grains. Tests for heterogeneity also suggested differing associations by tumor site and an inverse association for intake of fiber from fruit was observed but no association with risk of colon cancer. In a meta-analysis of prospective studies, no evidence of heterogeneity by anatomical site was observed for the association of intake of dietary fiber with risk (37). The latest WCRF meta-analysis assessing intake of fiber from fruit and colorectal cancer risk suggested a nonsignificant inverse association; however, tumor site heterogeneity was not reported possibly due to the absence of studies examining differences in risk by tumor site (2).

We observed that participants with a higher intake of fiber from whole grains had a lower risk of colorectal cancer. In the latest WCRF meta-analysis that included six prospective cohort studies, intake of whole grain foods (per 90 g/day) was associated with a 17% reduction in colorectal cancer risk (2). We observed a smaller inverse association for intake of whole grain foods (5% lower colorectal cancer risk per 50 g/day intake, which is equivalent to 9% lower per 90 g/day). In our subsample with 24-hour dietary assessment data, fiber from whole grains was infrequently consumed by most participants, which may introduce greater random measurement error and potential confounding if individuals who are consuming the most whole grains were more health conscious. Intake of total whole grains and fiber from whole grains has been suggested to reduce the risk of colorectal cancer through several potential mechanisms including reduced bowel transit time due to their high fiber content, greater stool bulk, presence of anticarcinogenic bioactive compounds (such as phenolic compounds and minerals), prevention of insulin resistance (2, 38), and SCFA production (6–9).

### Short-chain fatty acid genetic modifiers

SCFAs from fiber and whole grain microbiota fermentation have been thought to modulate colorectal cancer risk, with butyrate potentially having an anticarcinogenic effect (6–9). Besides diet, other factors can regulate the gut microbiome and the production of SCFA (39–41), including human genetic factors. In our analyses, we observed heterogeneity by host genetically predicted butyrate production; participants with a high PGS for butyrate production and consuming high amounts of whole grain starch had a lower risk of colorectal cancer, whereas there was a nonsignificant positive association between whole grain starch intake and risk of colorectal cancer for participants in the low butyrate PGS category. The nonsignificant positive association in the low butyrate PGS category contributed to the significant heterogeneity observed by whole grains starch intake; however, previous evidence has suggested that higher whole grain intake is inversely associated with colorectal cancer risk (2). We also did not observe evidence of significant heterogeneity for fiber from whole grains by the butyrate PGS, although the trends were similar to those observed for whole grain starch. This could be due lack of statistical power or possibly due to fiber intake in our analyses being calculated from NSP (from the UK Nutrient Databank); and resistant starch, which produces SCFA via microbiota fermentation in the colon (42), is not included in this calculation. When we performed sensitivity analyses using the larger UK Biobank cohort with the fiber from bread and cereals estimated from the few relevant dietary questions completed at recruitment (and previously shown to be inversely associated with colorectal cancer; ref. 43), we again observed significant heterogeneity by butyrate PGS, with an inverse association between intake of fiber from breads and cereals and colorectal cancer only observed in those in the high butyrate PGS category.

SCFAs, such as butyrate, have been shown to modulate cell histone deacetylases in the colorectum and function to reinforce cellular junctions, which may reduce low-grade inflammation, and lead to a lower risk of colorectal cancer (8, 44–46). Several studies have also shown that there is a lower abundance of butyrate-producing bacteria in the feces among individuals with colorectal cancer in comparison with control participants (9, 47). Some randomized controlled trials (RCT) have shown that a higher whole grain intake is associated with a higher abundance of SCFA-producing bacteria (5, 48, 49) and higher levels of SCFA in fecal samples compared with the control group (49, 50); however, other RCTs have found no differences in gut microbiota composition with higher whole grain intake (50, 51). Evidence from mouse models has suggested that fiber and whole grains are not protective against colorectal cancer risk without microbiota that can produce SCFA, such as butyrate (45, 52). Thus, the heterogeneity we observed for whole grain intake and colorectal cancer risk by butyrate PGS (which was shown to be associated with butyrate-producing bacteria) may underline the importance of butyrate production in reducing colorectal carcinogenesis, and further research in humans is needed to confirm these findings.

We also observed heterogeneity by the butyrate PGS for the association of total starch intake and colorectal cancer risk; participants in the low butyrate PGS category and consuming higher amounts of total starch had a higher risk of colorectal cancer, whereas no association was observed for those in the high butyrate PGS. Higher intake of certain sources of starch, such as potatoes, may promote a greater insulin response (53) and the butyrate PGS has been associated with an improved insulin response (21); however, it remains unclear if insulin response is the reason why there is a difference between butyrate PGS categories and starch intake, or these results may be due to chance as a result of multiple comparisons. Future research is needed to replicate these findings and better understand how SCFA production and human genetics may interact with dietary intake and influence the risk of colorectal cancer.

### Strengths and limitations

Strengths of this analysis include using repeated 24-hour dietary assessments to reduce random measurement error and the prospective nature of this study using linkage to health records, thus minimizing loss to follow-up. Due to the design of the 24-hour dietary assessments, we were able to look at sources and types of carbohydrates in detail to assess potential associations with colorectal cancer risk. Moreover, the UK Biobank collected detailed information from participants regarding socioeconomic and lifestyle factors and took standardized physical measurements, allowing us to adjust for these potential confounders in the analysis. In addition, genotyping was conducted in nearly the entire cohort allowing us to separate participants by host SCFA genetic factors.

Several limitations should be considered. With just over 1,000 cases of colorectal cancer in the main analyses, results may also be underpowered to detect modest associations. As well, numerous tests were performed, and if we correct for multiple testing, no significant findings would be observed, thus these results need to be interpreted cautiously. All self-reported dietary intake is subject to measurement error and some participants (∼39%) only completed two 24-hour dietary assessments and estimates will therefore be affected by random error, which biases the results toward the null. Although we adjusted for multiple confounders, unmeasured and residual confounding may still influence the associations observed. Reverse causality is also plausible due to colorectal cancer symptom onset potentially altering the consumption of specific foods. We did explore the potential for reverse causality in sensitivity analyses by removing the first 2 years of follow-up and estimates remained the same although with wider CIs; however, longer follow-up may still be required to account for the long latency period of this cancer. The UK Biobank is generally a healthy sample, with the subsample of participants completing the 24-hour dietary assessments being healthier, thus the results may not be generalizable to a wider population.

For analyses assessing effect modification by host genetically predicted SCFA production, the genetic scores utilized were derived from one sample of 952 individuals (21), limiting the power of these genetic variants in predicting differences in SCFA. Moreover, the butyrate PGS was found to predict the abundance of the PWY-5022 pathway, which was deemed to act as a proxy for butyrate production (21). However, this PGS has not been assessed with regard to the actual amount of butyrate produced or absorbed by the host and was found to poorly correlate with butyrate in fecal samples, but did associate with the abundance of butyrate-producing bacteria (*Eubacterium rectale* and *Roseburia intestinalis*) in fecal samples (21). Nevertheless, further research including genome-wide association studies is needed to confirm whether these genetic variants are reproducibly related to butyrate and propionate production. Moreover, our genetic analyses were restricted to white British individuals limiting the generalizability and power of these analyses. Considering this, these results need to be replicated using larger, more diverse samples to understand how genetics may interplay with carbohydrate intake, SCFA production, and colorectal cancer risk.

In summary, our results of this analysis of participants from the UK Biobank suggested that intakes of total carbohydrates, total sugars, non-free sugars, whole grain foods, and fiber from whole grains might be inversely associated with colorectal cancer risk. Some evidence of heterogeneity was observed by genetically predicted butyrate production, where the intake of starch from whole grains was inversely associated with colorectal cancer risk only in participants with high host genetically predicted butyrate synthesis. Although these results may support the possible importance of SCFA production in these associations, further research in other large prospective cohorts is needed to replicate these findings.

## Supplementary Material

Supplementary Material Supplementary Material
