# Supplementary material for: Prospective Analysis Reveals Associations between Carbohydrate Intakes, Genetic Predictors of Short-Chain Fatty Acid Synthesis, and Colorectal Cancer Risk
Source: Cancer Res. 2023 Apr 25;83(12):2066–76. doi: 10.1158/0008-5472.CAN-22-3755 (PMC10267681; doi:10.1158/0008-5472.CAN-22-3755)
Supplement: Supplementary Material [file can-22-3755_supplementary_material_suppsmst1-st8sf1-sf6.docx]

**Prospective analysis reveals associations between carbohydrate intakes, genetic predictors of short-chain fatty acid synthesis, and colorectal cancer risk**

Cody Z. Watling^1^, Rebecca K. Kelly^1^, Neil Murphy^2^, Marc Gunter^2^, Carmen Piernas^3^, Kathryn E. Bradbury^4^, Julie A. Schmidt^1,5^, Timothy J. Key^1^, Aurora Perez-Cornago^1^

**Author’s affiliations:**

^1^ Cancer Epidemiology Unit, Nuffield Department of Population Health, University of Oxford, Oxford, United Kingdom.

^2^ Nutrition and Metabolism Branch, International Agency for Research on Cancer, Lyon, France.

^3^ Nuffield Department of Primary Care, University of Oxford, Oxford, United Kingdom.

^4^ National Institute for Health Innovation, School of Population Health, The University of Auckland, Auckland, New Zealand.

^5^ Department of Clinical Epidemiology, Department of Clinical Medicine, Aarhus University and Aarhus University Hospital, Aarhus, Denmark

**Supplementary Materials**

[**Supplementary Methods** 4](#_Toc113971138)

[**Carbohydrate types and sources calculations** 4](#_Toc113971139)

[Carbohydrate calculations 4](#_Toc113971140)

[Fibre calculation 5](#_Toc113971141)

[**Covariates** 5](#_Toc113971142)

[**Polygenic score – Short-Chain Fatty Acids** 10](#_Toc113971143)

[**Supplementary Tables** 13](#_Toc113971144)

[**Supplementary Table 1.** Short chain fatty acid SNPs included in the polygenic risk scores derived from Sanna et al.^6^ 13](#_Toc113971145)

[**Supplementary Table 2.** Baseline characteristics of participants by lowest and highest quartile of intake of total carbohydrates, total sugars, non-free sugars, and free sugars. 14](#_Toc113971146)

[**Supplementary Table 3.** Baseline characteristics by lowest and highest quartile of intake of fibre intake from vegetables, fruits, and wholegrains 16](#_Toc113971147)

[**Supplementary Table 4.** Sequential adjustment hazard ratios and 95% confidence intervals for intake of carbohydrates sources with colorectal cancer risk (n=114,217) 18](#_Toc113971148)

[**Supplementary Table 5.** Multivariable adjusted hazard ratios and 95% confidence intervals for wholegrain and refined grain intake measured from total food weight of wholegrain and refined grain sources (n=114,217) 20](#_Toc113971149)

[**Supplementary Table 6**. Sequential adjustment hazard ratios and 95% confidence intervals for intake of fibre sources with colorectal cancer risk (n=114,217) 21](#_Toc113971150)

[**Supplementary Table 7.** Multivariable hazard ratios and 95% confidence intervals for carbohydrate and fibre sources with colorectal cancer risk restricting to participants who completed **≥3 24-hour dietary assessments** (N= 69,223; n cases = 734). 23](#_Toc113971151)

[**Supplementary Table 8.** Multivariable hazard ratios and 95% confidence intervals for carbohydrate and fibre sources with colorectal cancer risk **removing participants with <2 years of follow-up** (N=111,724; n cases = 978). 24](#_Toc113971152)

[**Supplementary Figures** 25](#_Toc113971153)

[**Supplementary Figure 1**. Dietary assessment for participants in the UK Biobank over time. 25](#_Toc113971154)

[**Supplementary Figure 2.** Flow chart of exclusion criteria for subsample of participants from the UK Biobank included in the main analyses. 26](#_Toc113971155)

[**Supplementary Figure 3.** Top food group contributors to types and sources of carbohydrates for participants in the 24-hour dietary assessment subsample (n=114,217) 27](#_Toc113971156)

[**Supplementary Figure 4.** Multivariable hazard ratios and 95% confidence intervals for carbohydrate and fibre sources with colorectal cancer risk **stratified by sex** 28](#_Toc113971157)

[**Supplementary Figure 5.** Multivariable hazard ratios and 95% confidence intervals for carbohydrate and fibre sources with colorectal cancer risk **stratified by body mass index (<27 kg/m^2^ vs. ≥27 kg/m^2^)** 29](#_Toc113971158)

[**Supplementary Figure 6.** Multivariable hazard ratios and 95% confidence intervals for carbohydrate and fibre sources with colorectal cancer risk **separated by tumour site (colon and rectal)** 30](#_Toc113971159)

# **Supplementary Methods**

## **Oxford WebQ 24-hour dietary assessment**

For the last 70,747 participants who were recruited between April 2009 and September 2010, the Oxford WebQ 24-hour dietary assessment was completed at their recruitment visit, while participants recruited before April 2009 did not complete a 24-hour dietary assessment at recruitment. Furthermore, between February 2011 and April 2012, a link to the 24-hour dietary assessment was emailed every 3-4 months for a total of four times to all participants in the UK Biobank who had provided a valid email address at recruitment (Supplementary Figure 1).

For the main analyses, we used a subsample of the UK Biobank who completed a minimum of two (maximum five) Oxford WebQ 24-hour dietary assessments in order to reduce random measurement error of intakes of carbohydrate and fibre as a result of the day to day variation in dietary intakes. During the follow-up period, the response rate to each follow-up 24-hour dietary assessment varied between 26% and 33% of participants with valid email addresses.

Nutrient intakes in the 24-hour dietary assessment were determined using the Nutrient Databank Food Composition table, which was matched to foods and beverages in the 24-hour dietary assessment to contain the nutrient composition of each item^1-3^.

Validation of the Oxford WebQ 24-hour dietary assessment has been conducted in a sample of 160 men and women with recovery biomarker measurements of protein (urinary nitrogen), potassium, a predictive biomarker of sugar (urinary fructose and sucrose), and total energy expenditure estimated by accelerometry^4^. When comparing the completion of two 24-hour dietary assessments with urinary sugar and energy measured from an accelerometer a correlation of 0.40 (95% CI: 0.24, 0.55) for sugar and 0.38 (95% CI: 0.21, 0.54) for energy and was observed, with higher correlations when additional 24-hour dietary assessments were completed^4^.

## Carbohydrate calculations

The following intakes of carbohydrates were automatically calculated from all food and beverages consumed in each Oxford WebQ 24-hour dietary assessment by multiplying consumption of nutrient composition: total carbohydrates, total sugars, free sugars, and total starch.

*Free sugars*

Free sugar intake was estimated from all monosaccharides and disaccharides added to foods by the manufacturer, plus sugars naturally present in honey, syrups, beverages, and fruit juices^2^.

*Non-free sugars*

Non-free sugars were calculated by subtracting free sugars from total sugars calculated for each 24-hour dietary assessment.

*Starch content of wholegrains*

The starch content of wholegrains was calculated from the intake of grams of starch from wholegrain bread, wholegrain pasta or brown rice, bran cereal, biscuit cereal (Weetabix), oatmeal (with or without sugar) and muesli.

*Starch content of refined grains*

The starch content of refined grains was calculated from the intake of grams of starch from white bread, other breads, white pasta or rice, other cereals, biscuits, cakes/pastries, savoury snacks, pizza, samosas, pakora crackers, grain dishes (added fat).

*Calculation of energy from carbohydrates types and sources*

Grams of carbohydrates, sugars, and starches were multiplied by 16.7 to obtain the kilojoules (kJ) of energy from the specific carbohydrate. Once this was determined from all 24-hour dietary assessments, each carbohydrate type and source was divided by the total amount of energy intake for the specific 24-hour dietary assessment for each participant to determine the percentage of energy from the carbohydrate for each individual 24-hour dietary assessment completed. From this, participants percentage of energy (or grams) from specific carbohydrate types and sources were averaged across all available 24-hour dietary assessments to determine the usual intake of each. Participants were then categorised into quartiles of averaged intake for each carbohydrate source.

## Fibre calculation

Fibre content was estimated for all foods in the 24-hour dietary assessments using the Englyst method^5^. Total fibre intake was derived from the fibre content of all foods reported to be consumed in the 24-hour dietary assessments.

*Fibre from vegetables*

Fibre from vegetables was calculated by adding together the grams of fibre from allium vegetables, leafy greens, raw salad, root vegetables, tomatoes, and the ‘other vegetables’ group.

*Fibre from fruits*

Fibre from fruits was calculated by combining the grams of fibre from apples/pears, berries, citrus fruits, dried fruit, other fruits, and stewed fruits.

*Fibre from vegetables and fruits*

Fibre from fruit and vegetables was calculated by combining the grams of fibre from vegetables and fruits described above.

*Fibre from wholegrains*

Fibre from wholegrains was calculated by adding together the grams of fibre from wholegrain bread, bran cereal, biscuit cereal (Weetabix), oatmeal, muesli, brown rice, and wholegrain pasta.

## **Colorectal cancer cases ascertainment**

Participants were first followed using National Health Service (NHS) Digital and Public Health England for participants from England and Wales and NHS Central Register for participants from Scotland. Follow-up was available until 29^th^ of February 2020 for England and Wales and and 31^st^ of January 2021 for Scotland from these databases. After these dates, participants from England and Scotland were followed using Hospital Episode Statistics admissions and Scottish Morbidity Records which covered time periods until September 2021 for England and July 31^st^, 2021 for Scottish participants. Participants information on primary and secondary cause for in-patient hospital admissions were used to determine an incident colorectal cancer diagnosis.

## **Covariates**

*Age at recruitment*

Participants provided their birthdate at the recruitment visit and age of participants were determined based on when they attended the recruitment visit. Participants were then categorised into age groups as <45, 45-49, 50-54, 55-59, 60-64, ≥65 years.

*Region*

Participants were grouped based on the recruitment centre they attended. A total of 10 regions were used corresponding approximately to the areas covered by the assessment centre: London (assessment centres: St Bartholomew’s Hospital, Hounslow, Croydon) Wales (assessment centres: Swansea, Wrexham, Cardiff), North-West England (assessment centres: Stockport, Manchester, Liverpool, Bury), North-East England (assessment centres: Newcastle, Middlesbrough), Yorkshire (assessment centres: Leeds, Sheffield), West Midlands (assessment centres: Stoke, Birmingham) East Midlands (assessment centre: Nottingham), South-East England (assessment centres: Oxford, Reading), South-West England (assessment centre: Bristol), Scotland (assessment centres: Glasgow, Edinburgh).

*Height*

Participants were grouped into seven sex-specific categories for height. For women categories were: <150, 150-154.9, 155-159.9, 160-164.9, 165-169.9, 170-174.9, ≥175 cm. For men categories were: <160, 160-164.9, 165-169.9, 170-174.9, 175-179.9, 180-184.9, ≥185 cm. From this, men and women were combined into one height variable with categories going from 1 to 8, and a missing category for participants with missing data (0.16% of participants).

*Body mass index (BMI)*

Both height and weight were measured at the baseline visit and were used to determine participant’s BMI. BMI was calculated by taking the participants measured weight in kilograms and dividing it by the participants squared standing height in metres. Individuals with missing data were coded into a missing category. Participants were categorised as follows: <20, 20-22.49,22.50-24.99, 25.00-27.49, 27.50-29.99, 30.00-32.49, 32.50-34.99, ≥35.00 kg/m^2^ and unknown/missing category (0.20%).

*Alcohol intake from the recruitment questionnaire*

Participants were asked on the baseline questionnaire at recruitment how often they drank alcohol with the possible responses being: “daily or almost daily”, “three or four times a week”, “once or twice a week”, “one to three times a month”, “special occasions only”, “never”, or “prefer not to answer”. Participants were also asked about their weekly and monthly intake of pints of beer, glasses of red wine, glasses of white wine/champagne, glasses of fortified wine, measures of spirits/liqueurs and glasses of other alcohol. A pint of beer was assumed to contain 20 grams of alcohol, and all other drinks contained 10 grams of alcohol, and then participants’ total weekly and monthly consumption of alcohol was calculated accordingly. If the participant reported ‘do not know’ or ‘prefer not to answer’ to one of these questions on weekly or monthly consumption, they were coded as missing, except for ‘other alcohol’, in which case we assigned them 0 grams from other alcohol. We used participants reported weekly consumption of alcohol, if this was unknown (due to the participant reporting ‘do not know’ or ‘prefer not to answer’ for one or more of the relevant questions, except for ‘other alcohol’) we used monthly consumption, if available. To get an estimated daily total, we divided weekly consumption by 7 (or monthly consumption by 30.44). Alcohol consumption was then categorised as none drinkers, <1 g/day, 1-9.99 g/day, 10-19.99 g/day, and ≥20 g/day, or unknown (0.36% of participants). For participants who had unknown grams/day of alcohol but who reported consuming alcohol intake on ‘special occasions’, we assigned them to the category of ‘<1 g/day’.

*Physical activity*

Physical activity was determined from questions on the touchscreen questionnaire which asked about walking, moderate physical activity, and vigorous physical activity. These were used to estimate excess metabolic equivalent (MET)-hours/week of physical activity during work and leisure time. For each of the three activity categories (walking, moderate physical activity and vigorous physical activity), participants were asked how many days in a typical week they did each of the activities for 10 minutes or more. For each category, participants who entered one or more days were then asked how many minutes they spent doing those activities on a typical day. For each activity category, the number of reported days was multiplied by the number of reported minutes on a typical day to generate duration of activity in minutes per week. Activity on a typical day of 1260 min per week (equivalent to an average of 3 hours per day) were truncated at 1260. Total MET values for each category from the International Physical Activity Questionnaire short form were: 3.3 for walking, 4.0 for moderate physical activity and 8.0 for vigorous physical activity. Excess MET values were therefore 2.3 for walking, 3.0 for moderate physical activity and 7.0 for vigorous physical activity. Excess MET-hours per week were calculated by multiplying the excess MET value for each activity by the duration of the activity in hours per week. Form this participants were categorised as low physical activity; 0-9.99, medium; 10-49.99, high; ≥50 excess MET-hours /week, and unknown/missing (1.81%).

*Townsend deprivation index*

Townsend deprivation index was based on the preceding national census output areas. Each participant was assigned a score in correspondence to the output area in which their postcode was located. From this, participants were spilt into quintiles from most deprived to least deprived and to a missing category when postcode information was not provided (0.12%).

*Smoking status*

Smoking was determined from questions from the recruitment questionnaire. Participants were asked “Do you smoke tobacco now?” and “in the past, how often have you smoked tobacco?” to determine their smoking status. Smokers were further divided based on how many cigarettes they said they smoked on average per day from the question ‘About how many cigarettes do you smoke on average each day?’. If participants did not respond to how many cigarettes they had on average each day, they were categorised into the missing group (0.22% of participants).

*Ethnicity*

The ethnicity of participants was determined from questions in the touchscreen questionnaire ‘What is your ethnic group?’. Options included: White, mixed, Asian or Asian British, Black or Black British, Chinese, and other ethnic group. From this, participants were grouped into five categories: White, mixed race or other, Asian or British Asian, and Black or Black British, or missing/unknown if information was not provided (0.33% of participants).

*Education*

For education, participants were asked ‘Which of the following qualification do you have?’ being able to select more than one. Possible answers were: College or University degree; A levels/AS levels or equivalent; O levels/GCSEs or equivalent; CSEs or equivalent; NVQ or HND or HNC or equivalent; Other professional qualifications example: nursing, teaching; None of the above; Prefer not to answer. We grouped participants into the following categories, based on their highest reported level of education: (College or University degree, vocational qualifications (other professional qualifications/NVQ or HND or HNC), optional national exams at ages 17 to 18 years (A levels/AS levels), national exams at age 16 years (O levels/GCSEs/CSEs), none of the above, unknown (prefer not to answer; (6.6% of participants)).

*Employment status*

Employment status at recruitment was assessed by asking participants ‘Which of the

following describes your current situation?’ in which they selected answers which were applicable to them including ‘In paid employment or self-employed’, ‘retired’, ‘looking after home and/or family’, ‘unable to work because of sickness or disability’, ‘unemployed’, ‘doing unpaid or voluntary work’, ‘full or part time student’, ‘none of the above’ or ‘prefer not to answer’. Participants were defined as being “in paid employment” if they responded they were in paid employment or self-employed, “retired” if they responded they were retired, and “not in paid employment” if they reported being unemployed, inability to work, being a student, or having caring responsibilities for their family. Finally, an unknown/missing category consisted of participants who did not respond, said they prefer not to answer, or answered none of the above to the options in the question (0.66% of participants).

*Diabetes status*

Participants diabetes status was determined from multiple variables collected at recruitment. Participants were asked ‘Has a doctor ever told you that you have diabetes?’ participants were classified as ‘yes’, ‘no’ or ‘unknown’ based on their response. We also used medication and blood levels of glycated hemoglobin (HbA1c) measured at recruitment to determine diabetes status. Specifically, participants who reported to using metformin or insulin at recruitment were considered as living with diabetes and included in the ‘yes’ category. Finally, if a participant had a measured glycated hemoglobin (HbA1c) of ≥ 48 mmol/mol at recruitment, they were defined as living with diabetes and included in the ‘yes’ category^6^. A total of 0.14% were categorised into the missing/unknown status category.

*Non-steroid anti-inflammatory drug use*

Non-steroid anti-inflammatory drug (NSAID) regular use was determined based on the medications reported at recruitment. Participants were categorised into three groups “Non-users”, “irregular NSAID users”, and “regular users of aspirin or ibuprofen”. Participants were categorised as “regular users of aspirin or ibuprofen” if they responded to taking ibuprofen or aspirin regularly in the touchscreen questionnaire completed at recruitment. Participants were categorised into irregular users if they responded to taking any NSAID at recruitment. If no use of NSAIDs were reported at recruitment, participants were categorised as non-users.

*Dietary variables*

*Red and processed meat intake*

Total red and processed meat intake was estimated from the touchscreen recruitment questionnaire from participants’ responses to how often they consumed beef, lamb/mutton, pork, and processed meat from the options of: ‘never, ‘<once a week’, ‘once a week’, ‘2-4 times a week’, ‘5-6 times a week’. The frequencies of consumption of beef, pork, lamb, and processed meat were summed by assigning participants intake as never a 0, less than once a week a 0.5, once a week a 1.0, 2-4 times a week a value of 3, and 5-6 times a week a value of 5.5. From this, intake of red and processed meat was categorised as < 2.0 times per week, 2.0-2.9 times per week, 3.0-3.9 times per week, and ≥4.0 times per week and an unknown/missing category if participants reported ‘do not know’ or ‘prefer not to say’ (0.41% of participants). This was estimated from the recruitment questionnaire rather than the 24-hour dietary assessment due to red and processed meat being consumed more episodically.

*Intake of fruit and vegetables*

Intake of fruit and vegetables were derived from the 24-hour dietary assessments. Intake of all fruits and vegetables in grams from: apples/pear, berries, citrus. dried fruit, other fruit, stewed fruit, allium vegetables, leafy greens, raw salad, root vegetables, tomatoes, other vegetables, and peas and corn were summed, and participants were categorised into quintiles from the total grams of intake.

*Menopausal hormone therapy*

For women, use of menopausal hormone therapy (MHT) was categorised as ‘current user’, ‘former user’ and ‘never user’ or ‘unknown/missing’ (0.22% of women) based on the questions asked about MHT use in the touchscreen questionnaire. All men were put into a separate category for all women-specific covariates. Women were asked ‘Have you ever used hormone replacement therapy?’ and if they answer yes they were also asked: ‘How old were you when you last used HRT?’. Women were asked to enter their age when they last used HRT or could choose ‘Still taking HRT’, or they could select ‘prefer not to answer’ or ‘do not know’. Women who selected prefer not to answer or don’t know were categorised as former users. Men were categorised into a separate category to retain them in the analyses.

*Menopausal status at recruitment*

Menopausal status was determined by multiple questions asked in the baseline questionnaire. Women were defined as being pre-menopausal if they:

- Answered ‘no’ to the question regarding having gone through menopause, or
- Reported they were ‘not sure’ or did not respond to if they had gone through menopause and:
  - Were <50 years of age, did not have a bilateral oophorectomy, and reported they were not using menopausal hormone therapy.
  - Were <50 years of age, reported they were menstruating today, and did not have a bilateral oophorectomy.

Women were defined as post-menopausal if they:

- Answered ‘yes’ to having gone through menopause
- Answered ‘not sure’ or did not answer if they had gone through menopause and:
  - were ≥55 years of age, or
  - had a bilateral oophorectomy

Women were defined as their menopausal status being unknown if:

- Answered ‘no’ to having gone through menopause and:
  - Did not answer no to using HRT, or
  - Did not answer no to having a bilateral oophorectomy, or
  - Did not answer no to having a hysterectomy, or
  - Were 50-54.9 years of age.

The unknown group consisted of 9.8% of participants. All men were categorised into a separate category to retain them in the analyses.

## **Polygenic score – Short-Chain Fatty Acids**

To calculate the polygenic score (PGS) for butyrate and propionate, we created a PGS previously reported by Sanna et al.^7^ in their genome-wide association study. For the butyrate PGS, we used 9 related single nucleotide polymorphisms (SNPs) that were associated with the pathway involved in 4-aminobuttanaoate (GABA) degradation (BioCyc ID: PWY-5022; variance explained = 16%; F-statistic=21) where butyrate is a product^7^. Abundance of the PWY-5022 pathway was associated with butyrate producing bacteria namely *Eubacterium rectale* and *Roseburia intestinalis.* For the propionate PGS, we used 3 related SNPs that were associated with fecal propionate levels (variance explained = 6.3%; F-statistic=21). The propionate SNPs were independent from the SNPs used for butyrate PWY-5022 abundance PGS. We calculated the PGS for butyrate and propionate according to the following calculation: PGS = (β_1_ × SNP_1_ + β _2_ × SNP_2_ + … β_9_ × SNP_9_) where β value was from the estimates of the previous genome wide association study^7^ and SNP represented the number of effect alleles for each SNPs (see table below for each SNP included in genetic score). In this subsample of the UK Biobank, no participants had any missing genotypes. Once the PGS were calculated for all participants, and appropriate exclusions were made (see main text), participants were separated by the median PGS (1.88 for butyrate PGS and 0.142 for propionate PGS) amongst eligible participants. Those in the >50 percentile were considered as high PGS whereas those in the ≤50^th^ percentile were considered as having a low PGS for both butyrate and propionate.

For these analyses including genetic factors, participants were excluded if they did not have genetic information (n=1,998) or reported they were not of white British ancestry (n=16,445). We also excluded participants with low call rates (<98% n=1,326), sex chromosome aneuploidy (n=66), and related individuals (kinship coefficient >0.0884; n=6,965), leaving a maximum of 87,417 participants and 909 cases of colorectal cancer in these analyses.

*Assessment of residual confounding comparing χ^2^ values*

To assess for residual confounding and the extent to which adjustment for confounders influenced the associations of intakes of carbohydrate types and sources with risk of colorectal cancer, the change of χ^2^ values were estimated using minimally adjusted models χ^2^ as the reference value. Comparing the change in χ^2^ values between minimally adjusted models (i.e., adjusting for age, sex, and region) to models with additional confounders (i.e., fully adjusted models) is a way to assess the extent to which additional confounders account for minimally adjusted associations between carbohydrates sources and types and colorectal cancer. As confounders are commonly measured imperfectly (e.g., due to changes in follow-up and measurement error) the proportional change in χ^2^ values from minimally adjusted models is a semiquantitative method for assessing residual confounding, as the addition of perfectly measured confounders in models are likely to reduce the χ^2^ value further and large reductions in χ^2^ values with the addition of imperfectly measured confounders may suggest residual confounding may explain the remaining observed association^8, 9^.

*Additional analysis in larger UK Biobank sample*

We also conducted a sensitivity analysis to see if there was heterogeneity in the association of intake of fibre from breads and cereals derived from the touchscreen questionnaire^10^ with colorectal cancer risk by SCFA PGS in the larger UK Biobank sample (n=343,621). This analyses were restricted to individuals that reported they were from white British ancestry and excluded participants with outliers for heterozygosity, low call rates (<98%), sex chromosome aneuploidy, and related individuals (kinship coefficient >0.0442). Due to the limited number of dietary questions asked at recruitment, sensitivity analyses further exploring SCFA heterogeneity were restricted to only this variable due to its similarity to the wholegrains variable determined for the 24-hour dietary assessments, and it was previously found to be associated with a lower risk of colorectal cancer^10^. The fibre from breads and cereals variable was calculated by the frequency and type of bread and cereals reportedly consumed by individuals at their recruitment visit. Specifically, participants were asked how many slices of bread or bowls of cereal they consumed per week as well as the type of bread and cereal they mainly consumed. For bread type, participants were asked what type they mainly ate, with possible answers being ‘white’, ‘brown’, ‘wholemeal or wholegrain’, ‘other type of bread’, ‘do not know’ or ‘prefer not to answer’. For cereal type, participants were asked what type they mainly ate, with possible answers being ‘bran cereal (e.g. All Bran, Branflakes)’, ‘biscuit cereal (e.g. Weetabix)’, oat cereal (e.g. Ready Brek, porridge)’ ‘muesli’, ‘other (e.g. Cornflakes, Frosties), ‘do not know’, ‘prefer not to answer’. The frequency of intake was converted from weekly consumption into a daily intake by dividing the reported intake by seven. From these responses, the non-starch polysaccharide content (in grams) of slices of bread and breakfast cereals were determined based on the frequency of consumption and types of bread or cereal reported in the touchscreen questionnaire. The amount of fibre in bread and cereal types were based on McCance and Widdowson’s The Composition of Foods (Seventh Summary Edition)^11^. Bread types were assigned the following fibre amounts per portion: 0.68 g for white bread, 1.26 g for brown bread, 1.80 g for wholemeal bread. Cereal types were assigned the following fibre amounts: 7.16 g for bran cereal, 2.92 g for biscuit cereal, 1.92 for oat cereal, 4.18g for muesli, and 0.54 g for other cereals. Participants who selected ‘do not know’ or ‘prefer not to answer’ for bread type or breakfast cereal type, but who reported their frequency of consumption of these food items, were assigned the average fibre content of these items. Participants who answered 0 or "Less than one" for slices of bread and bowls of breakfast cereal, respectively, were not asked which type of bread or cereal they usually consumed, and these participants were assigned a value of 0 g of fibre. Once the intake of fibre from bread and fibre from cereals were determined for all participants, these values were summed to estimate the fibre intake from breads and cereals. Greater detail of the calculation of fibre intake from these dietary responses at recruitment has been previously described elsewhere^12^. In these analyses we excluded all non-white British participants in addition to individuals who had a cancer diagnosis before recruitment, withdrew consent, or their genetic sex did not match their reported sex. We also adjusted all analyses for the first 10 principal components of ancestry. Adjustment for energy intake was not possible for this analysis due to the limited number of dietary questions asked in the recruitment questionnaire.

**References**

1. Swan G, Dodhia S, Farron-Wilson M, et al. Food composition data and public health. Nutrition Bulletin. 2015;40(3):223-6.

2. Perez-Cornago A, Pollard Z, Young H, et al. Description of the updated nutrition calculation of the Oxford WebQ questionnaire and comparison with the previous version among 207,144 participants in UK Biobank. Eur J Nutr. 2021;60(7):4019-30.

3. Piernas C, Perez-Cornago A, Gao M, et al. Describing a new food group classification system for UK biobank: analysis of food groups and sources of macro- and micronutrients in 208,200 participants. Eur J Nutr. 2021;60(5):2879-90.

4. Greenwood DC, Hardie LJ, Frost GS, et al. Validation of the Oxford WebQ online 24-hour dietary questionnaire using biomarkers. Am J Epidemiol. 2019;188(10):1858-67.

5. Englyst HN, Cummings JH. Improved method for measurement of dietary fiber as non-starch polysaccharides in plant foods. J Assoc Off Anal Chem. 1988;71(4):808-14.

6. Diabetes UK. Diagnostic criteria for diabetes [Accessed 2022 March 20]. Available from: <https://www.diabetes.org.uk/professionals/position-statements-reports/diagnosis-ongoing-management-monitoring/new_diagnostic_criteria_for_diabetes>.

7. Sanna S, van Zuydam NR, Mahajan A, et al. Causal relationships among the gut microbiome, short-chain fatty acids and metabolic diseases. Nat Genet. 2019;51(4):600-5.

8. Floud S, Balkwill A, Moser K, et al. The role of health-related behavioural factors in accounting for inequalities in coronary heart disease risk by education and area deprivation: prospective study of 1.2 million UK women. BMC Med. 2016;14(1):145.

9. Feng Q, Kim JH, Omiyale W, et al. Raw and Cooked Vegetable Consumption and Risk of Cardiovascular Disease: A Study of 400,000 Adults in UK Biobank. Frontiers in Nutrition. 2022;9.

10. Bradbury KE, Murphy N, Key TJ. Diet and colorectal cancer in UK Biobank: a prospective study. Int J Epidemiol. 2019;49(1):246-58.

11. Finglas PM RM, Pinchen HM, et al. McCance and Widdowson’s The Compostion of Foods, Seventh Summary Edition. Cambridge: Royal Society of Chemistry. 2015.

12. Bradbury KE, Young HJ, Guo W, et al. Dietary assessment in UK Biobank: an evaluation of the performance of the touchscreen dietary questionnaire. J Nutr Sci. 2018;7:e6.

# **Supplementary Tables**

## **Supplementary Table 1.** Short chain fatty acid SNPs included in the polygenic risk scores derived from Sanna et al.^7^

| SNP (rs ID) | Effect Allele | Effect allele frequency | Beta (95% CI) | Standard Error | R^2^ | Effect allele frequency in UKB |
| --- | --- | --- | --- | --- | --- | --- |
| Butyrate (PWY-5022 pathway) score | | | | | | |
| rs9423658 | C | 0.86 | 0.33 (0.19,0.48) | 0.07653061 | <0.1 | 0.84 |
| rs881390 | C | 0.11 | 0.40 (0.23,0.57) | 0.08673469 | <0.1 | 0.12 |
| rs2089222 | A | 0.04 | 0.56 (0.32,0.79) | 0.11734694 | <0.1 | 0.04 |
| rs9904981 | G | 0.77 | 0.25 (0.14,0.36) | 0.05612245 | <0.1 | 0.80 |
| rs10483112 | T | 0.03 | 0.59 (0.34,0.84) | 0.12755102 | <0.1 | 0.03 |
| rs12994030 | T | 0.27 | 0.24 (0.14,0.34) | 0.05102041 | <0.1 | 0.28 |
| rs2056208 | T | 0.24 | 0.24 (0.14,0.34) | 0.05612245 | <0.1 | 0.27 |
| rs10019739 | C | 0.27 | 0.24 (0.13,0.34) | 0.05102041 | <0.1 | 0.27 |
| rs7743827 | G | 0.80 | 0.27 (0.15,0.38) | 0.05612245 | <0.1 | 0.80 |
| Propionate score | | | | | | |
| rs7142308 | G | 0.61 | 0.24 (0.14,0.34) | 0.05102041 | <0.1 | 0.63 |
| rs12050534 | C | 0.14 | 0.31 (0.18,0.44) | 0.06632653 | <0.1 | 0.16 |
| rs1400566 | G | 0.45 | -0.22 (-0.31, -0.12) | 0.05102041 | <0.1 | 0.56 |

Abbreviations: SNPs, single nucleotide polymorphisms; UKB, UK Biobank.

| **Supplementary Table 2.** Baseline characteristics of participants by lowest and highest quartile of intake of total carbohydrates, total sugars, non-free sugars, and free sugars. | | | | | | | | | | | |
| --- | --- | --- | --- | --- | --- | --- | --- | --- | --- | --- | --- |
|  | **Total carbohydrates** | |  | **Total sugars** | |  | **Total non-free sugar** | |  | **Total free sugar** | |
|  | **Q1** | **Q4** |  | **Q1** | **Q4** |  | **Q1** | **Q4** |  | **Q1** | **Q4** |
| No. of participants | 28555 | 28553 |  | 28555 | 28554 |  | 28555 | 28554 |  | 28555 | 28554 |
| Intake of carbohydrate of interest ^a^ | 39.9 (4.6) | 58.3 (3.5) |  | 16.5 (2.7) | 32.9 (4.1) |  | 6.8 (1.6) | 20.5 (4.1) |  | 5.8 (1.7) | 17.9 (3.7) |
| Sex - Male, N (%) | 13810 (48.4%) | 12027 (42.1%) |  | 14683 (51.4%) | 10706 (37.5%) |  | 17274 (60.5%) | 8498 (29.8%) |  | 10586 (37.1%) | 15225 (53.3%) |
| Age at recruitment - years | 55.9 (7.7) | 55.8 (8.0) |  | 54.9 (7.9) | 56.7 (7.7) |  | 54.1 (8.0) | 57.3 (7.4) |  | 56.2 (7.6) | 55.4 (8.1) |
| Body mass index | 27.1 (4.6) | 26.4 (4.6) |  | 27.2 (4.7) | 26.4 (4.5) |  | 27.3 (4.6) | 26.4 (4.6) |  | 26.9 (4.8) | 26.7 (4.5) |
| Height | 170.1 (9.1) | 168.7 (9.1) |  | 170.3 (9.2) | 168.2 (9.0) |  | 171.6 (9.1) | 167.1 (8.7) |  | 168.2 (9.0) | 170.5 (9.2) |
| Physical activity - High, N (%) | 4573 (16.0%) | 5353 (18.7%) |  | 4213 (14.8%) | 5839 (20.4%) |  | 4217 (14.8%) | 5785 (20.3%) |  | 4755 (16.7%) | 5143 (18.0%) |
| Townsend deprivation index | |  |  |  |  |  |  |  |  |  |  |
| Q1 - Most affluent | 5998 (21.0%) | 6015 (21.1%) |  | 5941 (20.8%) | 6262 (21.9%) |  | 5775 (20.2%) | 6300 (22.1%) |  | 6014 (21.1%) | 6072 (21.3%) |
| Q5 - Most deprived | 4991 (17.5%) | 4671 (16.4%) |  | 5127 (18.0%) | 4539 (15.9%) |  | 5316 (18.6%) | 4397 (15.4%) |  | 4900 (17.2%) | 4898 (17.2%) |
| In paid employment, N (%) | 18277 (64.0%) | 17397 (60.9%) |  | 19121 (67.0%) | 16576 (58.1%) |  | 19609 (68.7%) | 16203 (56.7%) |  | 17683 (61.9%) | 17896 (62.7%) |
| University/college degree, N (%) | 21372 (74.8%) | 20510 (71.8%) |  | 21002 (73.5%) | 20603 (72.2%) |  | 20469 (71.7%) | 20949 (73.4%) |  | 21030 (73.6%) | 20305 (71.1%) |
| Ethnicity, N (%) |  |  |  |  |  |  |  |  |  |  |  |
| White | 27804 (97.4%) | 27106 (94.9%) |  | 27592 (96.6%) | 27344 (95.8%) |  | 27521 (96.4%) | 27479 (96.2%) |  | 27578 (96.6%) | 27309 (95.6%) |
| Mixed race/ Other | 282 (1.0%) | 422 (1.5%) |  | 335 (1.2%) | 363 (1.3%) |  | 340 (1.2%) | 350 (1.2%) |  | 339 (1.2%) | 359 (1.3%) |
| Asian or British Asian | 190 (0.7%) | 600 (2.1%) |  | 357 (1.3%) | 395 (1.4%) |  | 293 (1.0%) | 420 (1.5%) |  | 415 (1.5%) | 357 (1.3%) |
| Black or Black British | 170 (0.6%) | 340 (1.2%) |  | 165 (0.6%) | 363 (1.3%) |  | 294 (1.0%) | 224 (0.8%) |  | 134 (0.5%) | 419 (1.5%) |
| Smoking - Never, N (%) | 13467 (47.2%) | 18486 (64.7%) |  | 14474 (50.7%) | 17614 (61.7%) |  | 14450 (50.6%) | 17645 (61.8%) |  | 15484 (54.2%) | 16500 (57.8%) |
| Diabetes - Yes, N(%) | 1215 (4.3%) | 989 (3.5%) |  | 1677 (5.9%) | 699 (2.4%) |  | 1043 (3.7%) | 1041 (3.6%) |  | 1893 (6.6%) | 611 (2.1%) |
| NSAID - regular user, N(%) | 7553 (26.5%) | 6817 (23.9%) |  | 7636 (26.7%) | 7085 (24.8%) |  | 7509 (26.3%) | 6794 (23.8%) |  | 7290 (25.5%) | 7274 (25.5%) |
| Postmenopausal at recruitment | 9880 (67.0%) | 11177 (67.7%) |  | 8297 (59.8%) | 13022 (73.0%) |  | 6044 (53.6%) | 15230 (76.0%) |  | 12514 (69.7%) | 8496 (63.8%) |
| Menopausal hormone therapy use - Current, N (%) | 1378 (9.3%) | 1234 (7.5%) |  | 1145 (8.3%) | 1516 (8.5%) |  | 933 (8.3%) | 1680 (8.4%) |  | 1502 (8.4%) | 1102 (8.3%) |
| **Diet variables** |  |  |  |  |  |  |  |  |  |  |  |
| Alcohol intake - g/day | 24.7 (21.1) | 9.6 (11.9) |  | 21.4 (19.3) | 11.6 (14.2) |  | 23.4 (21.8) | 10.7 (11.9) |  | 15.8 (16.1) | 16.8 (19.2) |
| Red and processed meat intake - 4.00+ times/wk, N (%) | 12961 (45.4%) | 8698 (30.5%) |  | 13033 (45.6%) | 8776 (30.7%) |  | 14489 (50.7%) | 7406 (25.9%) |  | 10084 (35.3%) | 12123 (42.5%) |
| Vegetable and fruit intake - g/day | 325.7 (197.8) | 453.2 (255.6) |  | 271.9 (161.2) | 506.6 (268.9) |  | 199.8 (117.5) | 590.4 (246.1) |  | 449.1 (253.7) | 318.7 (197.8) |
| Total carbohydrate intake - % energy | 39.9 (4.6) | 58.3 (3.5) |  | 43.0 (6.9) | 55.7 (5.5) |  | 44.8 (7.4) | 54.1 (6.3) |  | 46.5 (8.2) | 52.7 (6.5) |
| Total sugar intake - % energy | 19.0 (4.6) | 30.2 (6.2) |  | 16.5 (2.7) | 32.9 (4.1) |  | 19.5 (5.6) | 30.4 (5.5) |  | 20.4 (6.3) | 29.3 (5.7) |
| Total starch intake - % energy | 20.8 (4.9) | 28.1 (5.6) |  | 26.5 (6.3) | 22.7 (5.0) |  | 25.3 (6.0) | 23.6 (5.5) |  | 26.1 (6.4) | 23.3 (5.1) |
| Total fibre intake - g/day | 15.4 (5.0) | 19.9 (6.1) |  | 16.1 (5.2) | 19.2 (6.2) |  | 14.8 (4.7) | 20.7 (6.1) |  | 18.6 (6.0) | 16.5 (5.3) |
| Total wholegrain fibre - g/day | 2.6 (2.5) | 4.5 (3.3) |  | 3.1 (3.0) | 3.9 (3.0) |  | 2.7 (2.8) | 4.2 (3.0) |  | 3.7 (3.1) | 3.3 (2.9) |
| Total energy intake – kJ/day | 8842 (2137) | 8186 (1919) |  | 8822 (2082) | 8238(1978) |  | 9215 (2153) | 7875 (1816) |  | 8098 (1919) | 8928 (2103) |
| Values are mean (SD) unless otherwise indicated.  ^a^ values represent % of energy from carbohydrates, sugars, non-free sugars, and free sugars. | | | | | | | | | | | |
| Abbreviations: g, grams; g/day, grams per day; kJ, kilojoules; NSAID, non-steroid anti-inflammatory drug; N, Number of participants; Q, quartile; wk, week. | | | | | | | | | | | |

| **Supplementary Table 3.** Baseline characteristics by lowest and highest quartile of intake of fibre intake from vegetables, fruits, and wholegrains | | | | | | | | |
| --- | --- | --- | --- | --- | --- | --- | --- | --- |
|  | **Fibre from vegetables** | |  | **Fibre from fruit** | |  | **Fibre from wholegrains** | |
|  | **Q1** | **Q4** |  | **Q1** | **Q4** |  | **Q1** | **Q4** |
| No. of participants | 28555 | 28554 |  | 28594 | 28553 |  | 28655 | 27817 |
| Intake of carbohydrate of interest ^a^ | 0.7 (0.5) | 6.1 (2.1) |  | 0.6 (0.4) | 5.6 (1.9) |  | 0.4 (0.5) | 7.9 (2.3) |
| Sex - Male, N (%) | 16050 (56.2%) | 10264 (35.9%) |  | 15269 (53.4%) | 11148 (39.0%) |  | 12484 (43.6%) | 14640 (52.6%) |
| Age at recruitment - years | 54.5 (7.9) | 56.9 (7.7) |  | 54.1 (7.9) | 57.4 (7.4) |  | 55.0 (7.9) | 56.6 (7.7) |
| Body mass index | 27.2 (4.7) | 26.5 (4.6) |  | 27.2 (4.7) | 26.4 (4.6) |  | 27.3 (4.9) | 26.0 (4.3) |
| Height | 170.7 (9.3) | 168.4 (8.9) |  | 170.4 (9.2) | 168.8 (9.1) |  | 168.9 (9.1) | 170.8 (9.2) |
| Physical activity - High, N (%) | 4220 (14.8%) | 6085 (21.3%) |  | 4202 (14.7%) | 5939 (20.8%) |  | 4656 (16.2%) | 5122 (18.4%) |
| Townsend deprivation index |  |  |  |  |  |  |  |  |
| Q1 - Most affluent | 5934 (20.8%) | 6075 (21.3%) |  | 5978 (20.9%) | 6226 (21.8%) |  | 5740 (20.0%) | 6393 (23.0%) |
| Q5 - Most deprived | 5082 (17.8%) | 4557 (16.0%) |  | 5074 (17.7%) | 4481 (15.7%) |  | 5413 (18.9%) | 4048 (14.6%) |
| In paid employment, N (%) | 19362 (67.8%) | 16519 (57.9%) |  | 19347 (67.7%) | 16353 (57.3%) |  | 18813 (65.7%) | 16465 (59.2%) |
| University/college degree, N (%) | 20146 (70.6%) | 21094 (73.9%) |  | 19869 (69.5%) | 21732 (76.1%) |  | 20157 (70.3%) | 21289 (76.5%) |
| Ethnicity, N (%) |  |  |  |  |  |  |  |  |
| White | 27368 (95.8%) | 27618 (96.7%) |  | 27649 (96.7%) | 27407 (96.0%) |  | 27334 (95.4%) | 27185 (97.7%) |
| Mixed race/ Other | 321 (1.1%) | 344 (1.2%) |  | 299 (1.0%) | 398 (1.4%) |  | 413 (1.4%) | 216 (0.8%) |
| Asian or British Asian | 446 (1.6%) | 290 (1.0%) |  | 290 (1.0%) | 397 (1.4%) |  | 456 (1.6%) | 207 (0.7%) |
| Black or Black British | 314 (1.1%) | 201 (0.7%) |  | 256 (0.9%) | 255 (0.9%) |  | 336 (1.2%) | 131 (0.5%) |
| Never smokers, N (%) | 16246 (56.9%) | 16025 (56.1%) |  | 15209 (53.2%) | 17046 (59.7%) |  | 15161 (52.9%) | 16973 (61.0%) |
| Alcohol intake - g/day | 17.3 (18.5) | 15.5 (16.6) |  | 20.4 (20.4) | 13.1 (14.2) |  | 19.0 (20.4) | 14.8 (14.6) |
| Diabetes - Yes, N (%) | 1224 (4.3%) | 1076 (3.8%) |  | 1064 (3.7%) | 1135 (4.0%) |  | 1089 (3.8%) | 1047 (3.8%) |
| NSAID - regular user, N (%) | 7411 (26.0%) | 7066 (24.7%) |  | 7547 (26.4%) | 6904 (24.2%) |  | 7576 (26.4%) | 6543 (23.5%) |
| Postmenopausal at recruitment, N (%) | 7534 (60.3%) | 13220 (72.3%) |  | 7453 (56.0%) | 13191 (75.8%) |  | 10190 (63.0%) | 9280 (70.5%) |
| Menopausal hormone therapy use - Current, N (%) | 1079 (8.6%) | 1498 (8.2%) |  | 1069 (8.0%) | 1459 (8.4%) |  | 1397 (8.6%) | 943 (7.2%) |
| **Diet variables** |  |  |  |  |  |  |  |  |
| Red and processed meat intake - 4+ times/wk, N (%) | 12362 (43.3%) | 9412 (33.0%) |  | 13322 (46.6%) | 8777 (30.7%) |  | 12603 (44.0%) | 9811 (35.3%) |
| Vegetable and fruit intake - grams | 2.8 (1.9) | 9.7 (3.5) |  | 2.9 (2.0) | 9.6 (3.6) |  | 5.1 (3.5) | 6.4 (3.6) |
| Total carbohydrate intake - % energy | 49.7 (7.5) | 49.3 (7.7) |  | 46.1 (7.6) | 52.8 (6.8) |  | 46.8 (8.2) | 51.7 (6.4) |
| Total sugar intake - % energy | 23.5 (6.7) | 25.6 (6.7) |  | 20.3 (5.9) | 29.1 (6.1) |  | 23.2 (7.2) | 25.1 (5.9) |
| Total starch intake - % energy | 26.1 (5.8) | 23.5 (5.7) |  | 25.8 (6.1) | 23.6 (5.4) |  | 23.6 (6.3) | 26.5 (5.0) |
| Total fibre intake - g/day | 14.5 (4.6) | 22.2 (5.8) |  | 14.3 (4.5) | 22.2 (5.7) |  | 14.4 (4.8) | 22.1 (5.4) |
| Total wholegrain fibre - g/day | 3.4 (3.0) | 3.9 (3.1) |  | 2.9 (2.8) | 4.3 (3.2) |  | 0.4 (0.5) | 7.9 (2.3) |
| Total energy intake – kJ/day | 8389 (1975) | 8866 (1975) |  | 8502 (1997) | 8786 (1944) |  | 8363 (2005) | 9025 (1912) |
| Values are mean (SD) unless otherwise indicated.  ^a^ values represent grams of fibre from vegetables, fruits, and wholegrains. | | | | | | | | |
| Abbreviations: g, grams; g/d, grams per day; kJ, kilojoules; NSAID, non-steroid anti-inflammatory drug; N, Number of participants; Q, quartile; wk, week. | | | | | | | | |

| **Supplementary Table 4.** Sequential adjustment hazard ratios and 95% confidence intervals for intake of carbohydrates sources with colorectal cancer risk (n=114,217) | | | | | | | | |
| --- | --- | --- | --- | --- | --- | --- | --- | --- |
|  | **Q1** | **Q2** | **Q3** | **Q4** | **Per 5% energy increment** | ***χ*^2^** | ***χ*^2^ – %**  **change** | ***P-trend*** |
| **Total carbohydrates** |  |  |  |  |  |  |  |  |
| Minimally-adjusted* | 1 (ref) | **0.83 (0.71 - 0.96)** | **0.82 (0.70 - 0.96)** | **0.71 (0.60 - 0.83)** | **0.91 (0.88 - 0.95)** | 22.29 |  | <0.001 |
| Multivariable-adjusted** | 1 (ref) | 0.87 (0.74 - 1.01) | 0.89 (0.76 - 1.05) | **0.79 (0.66 - 0.95)** | **0.94 (0.90 - 0.98)** | 9.44 | -57.6% | 0.002 |
| + BMI | 1 (ref) | 0.87 (0.75 - 1.02) | 0.90 (0.77 - 1.07) | **0.81 (0.68 - 0.97)** | **0.94 (0.90 - 0.98)** | 7.98 | -64.2% | 0.005 |
| + Processed/red meat & total vegetable and fruit intake | 1 (ref) | 0.88 (0.75 - 1.03) | 0.92 (0.78 - 1.08) | 0.83 (0.70 - 1.00) | **0.95 (0.91 - 0.99)** | 5.88 | -73.6% | 0.015 |
|  |  |  |  |  |  |  |  |  |
| **Total sugar** |  |  |  |  |  |  |  |  |
| Minimally-adjusted* | 1 (ref) | **0.81 (0.69 - 0.94)** | **0.74 (0.64 - 0.87)** | **0.67 (0.57 - 0.79)** | **0.90 (0.86 - 0.94)** | 22.14 |  | <0.001 |
| Multivariable-adjusted** | 1 (ref) | **0.84 (0.72 - 0.98)** | **0.79 (0.67 - 0.93)** | **0.73 (0.62 - 0.87)** | **0.92 (0.88 - 0.97)** | 11.42 | -48.4% | 0.001 |
| + BMI | 1 (ref) | **0.85 (0.72 - 0.99)** | **0.80 (0.68 - 0.94)** | **0.74 (0.63 - 0.88)** | **0.93 (0.88 - 0.97)** | 10.33 | -53.3% | 0.001 |
| + Processed/red meat & total vegetable and fruit intake | 1 (ref) | **0.84 (0.72 - 0.99)** | **0.80 (0.68 - 0.94)** | **0.75 (0.63 - 0.90)** | **0.93 (0.88 - 0.98)** | 7.58 | -65.8% | 0.006 |
|  |  |  |  |  |  |  |  |  |
| **Free sugars** |  |  |  |  |  |  |  |  |
| Minimally-adjusted* | 1 (ref) | 1.09 (0.93 - 1.28) | 0.98 (0.84 - 1.16) | 0.90 (0.76 - 1.07) | 0.97 (0.91 - 1.03) | 1.13 |  | 0.29 |
| Multivariable-adjusted** | 1 (ref) | 1.10 (0.93 - 1.28) | 0.99 (0.84 - 1.16) | 0.89 (0.76 - 1.06) | 0.96 (0.91 - 1.02) | 1.45 | +28.3% | 0.23 |
| + BMI | 1 (ref) | 1.10 (0.94 - 1.29) | 0.99 (0.84 - 1.17) | 0.90 (0.76 - 1.07) | 0.97 (0.91 - 1.03) | 1.27 | +11.8% | 0.26 |
| + Processed/red meat & total vegetable and fruit intake | 1 (ref) | 1.08 (0.92 - 1.27) | 0.97 (0.82 - 1.14) | 0.87 (0.73 - 1.04) | 0.95 (0.90 - 1.02) | 2.24 | +97.7% | 0.14 |
|  |  |  |  |  |  |  |  |  |
| **Non-free sugars** |  |  |  |  |  |  |  |  |
| Minimally-adjusted* | 1 (ref) | **0.82 (0.71 - 0.96)** | **0.72 (0.62 - 0.85)** | **0.68 (0.57 - 0.80)** | **0.87 (0.82 - 0.92)** | 23.00 |  | <0.001 |
| Multivariable-adjusted** | 1 (ref) | 0.87 (0.74 - 1.01) | **0.79 (0.67 - 0.93)** | **0.77 (0.65 - 0.92)** | **0.91 (0.86 - 0.97)** | 9.34 | -59.4% | 0.002 |
| + BMI | 1 (ref) | 0.87 (0.75 - 1.02) | **0.79 (0.67 - 0.94)** | **0.78 (0.65 - 0.93)** | **0.92 (0.86 - 0.97)** | 8.57 | -62.7% | 0.003 |
| + Processed/red meat | 1 (ref) | 0.87 (0.75 - 1.02) | **0.80 (0.68 - 0.94)** | **0.79 (0.66 - 0.94)** | **0.92 (0.86 - 0.98)** | 7.60 | -67.0% | 0.006 |
|  |  |  |  |  |  |  |  |  |
|  | **Q1** | **Q2** | **Q3** | **Q4** | **Per 5% energy increment** | ***χ*^2^** | ***χ*^2^ – % change** | ***P-trend*** |
| **Total starch** |  |  |  |  |  |  |  |  |
| Minimally-adjusted* | 1 (ref) | 0.91 (0.78 - 1.07) | 0.93 (0.80 - 1.09) | 0.92 (0.78 - 1.08) | 0.98 (0.93 - 1.03) | 0.80 |  | 0.37 |
| Multivariable-adjusted** | 1 (ref) | 0.93 (0.79 - 1.09) | 0.97 (0.83 - 1.14) | 0.98 (0.83 - 1.16) | 1.00 (0.95 - 1.06) | 0.00 | -100.0% | 0.99 |
| + BMI | 1 (ref) | 0.93 (0.80 - 1.09) | 0.98 (0.83 - 1.15) | 0.99 (0.84 - 1.17) | 1.00 (0.95 - 1.06) | 0.02 | -97.9% | 0.90 |
| + Processed/red meat & total vegetable and fruit intake | 1 (ref) | 0.92 (0.79 - 1.08) | 0.96 (0.82 - 1.13) | 0.98 (0.82 - 1.16) | 1.00 (0.94 - 1.06) | 0.00 | -99.6% | 0.96 |
|  |  |  |  |  |  |  |  |  |
| **Wholegrain starch** |  |  |  |  |  |  |  |  |
| Minimally-adjusted* | 1 (ref) | 0.88 (0.75 - 1.03) | **0.84 (0.71 - 0.98)** | **0.77 (0.66 - 0.91)** | **0.89 (0.83 - 0.95)** | 10.74 |  | 0.001 |
| Multivariable-adjusted** | 1 (ref) | 0.89 (0.76 - 1.05) | 0.87 (0.74 - 1.02) | **0.83 (0.70 - 0.97)** | **0.92 (0.85 - 0.99)** | 5.26 | -51.1% | 0.022 |
| + BMI | 1 (ref) | 0.90 (0.77 - 1.06) | 0.88 (0.75 - 1.04) | 0.85 (0.72 - 1.00) | 0.93 (0.87 - 1.00) | 3.73 | -65.3% | 0.053 |
| + Processed/red meat & total vegetable and fruit intake | 1 (ref) | 0.90 (0.77 - 1.06) | 0.89 (0.76 - 1.05) | 0.86 (0.73 - 1.02) | 0.94 (0.87 - 1.01) | 2.90 | -73.0% | 0.089 |
|  |  |  |  |  |  |  |  |  |
| **Refined grain starch** |  |  |  |  |  |  |  |  |
| Minimally-adjusted* | 1 (ref) | 0.93 (0.80 - 1.10) | 1.07 (0.91 - 1.25) | 1.09 (0.93 - 1.29) | 1.04 (0.99 - 1.09) | 2.10 |  | 0.15 |
| Multivariable-adjusted** | 1 (ref) | 0.92 (0.78 - 1.08) | 1.05 (0.89 - 1.23) | 1.09 (0.92 - 1.28) | 1.04 (0.99 - 1.09) | 2.06 | -1.6% | 0.15 |
| + BMI | 1 (ref) | 0.92 (0.78 - 1.08) | 1.04 (0.89 - 1.22) | 1.08 (0.91 - 1.27) | 1.03 (0.98 - 1.08) | 1.77 | -15.4% | 0.18 |
| + Processed/red meat & total vegetable and fruit intake | 1 (ref) | 0.91 (0.77 - 1.06) | 1.02 (0.87 - 1.20) | 1.05 (0.89 - 1.25) | 1.03 (0.98 - 1.08) | 1.06 | -49.7% | 0.30 |
| *Minimally-adjusted model stratified for sex, age at recruitment (<45, 45-49, 50-54, 55-59, 60-64, ≥65 years) and adjusted for region (North-West England, North-Eastern England, Yorkshire & the Humber, West Midlands, East Midlands, South-East England, South-West England, London, Wales, and Scotland)  **Multivariable adjusted model further adjusting for height, physical activity, Townsend deprivation index, education, employment, smoking, alcohol consumption measured at recruitment, ethnicity, diabetes status, non-steroidal anti-inflammatory drug use, energy intake, female specific covariates: menopausal hormone therapy use and menopausal status.  + BMI: further adjusting for BMI categories.  + processed/red meat & total vegetable and fruit intake: further adjusting for red and processed meat consumption reported at recruitment and fruit and vegetables intake measured by averaged 24-hour dietary assessments.  χ^2^ and p-trend represent improvement of fit obtained from likelihood ratio tests for including the carbohydrate type/source (modelled as a 5% energy increase).  χ^2^ - % change calculated from the percentage difference in χ^2^ value using the minimally adjusted χ^2^ value as the reference group.  Abbreviations: BMI, body mass index; Q, quartile; ref, reference group. | | | | | | | | |

| **Supplementary Table 5.** Multivariable adjusted hazard ratios and 95% confidence intervals for wholegrain and refined grain intake measured from total food weight of wholegrain and refined grain sources (n=114,217) | | | |
| --- | --- | --- | --- |
| **Wholegrains** | **N/Cases** | **g/day** | **HR (95% CI)** |
| Q1 | 28,927 / 329 | 8.71 (10.8) | 1 (ref) |
| Q2 | 28,184 / 301 | 52.0 (12.3) | 0.93 (0.79 - 1.08) |
| Q3 | 28,604 / 294 | 101.2 (17.2) | 0.88 (0.74- 1.03) |
| Q4 | 28,502 / 269 | 204.5 (61.8) | **0.79 (0.67 - 0.94)** |
| Per 50 g/day | - | - | **0.95 (0.91 - 0.98)** |
| *p-value* | - | - | 0.004 |
|  |  |  |  |
| **Refined grains** | **N/Cases** | **g/day** | **HR (95% CI)** |
| Q1 | 28,560 / 299 | 54.9 (25.2) | 1 (ref) |
| Q2 | 28,565 / 290 | 120.5 (16.3) | 0.95 (0.81 - 1.12) |
| Q3 | 28,546 / 302 | 180.6 (19.5) | 1.00 (0.85 - 1.19) |
| Q4 | 28,546 / 302 | 293.5 (70.8) | 1.05 (0.87 - 1.26) |
| Per 50 g/day | - | - | 1.02 (0.98 - 1.05) |
| *p-value* | - | - | 0.366 |
| Models stratified for sex and age at recruitment, and further adjusted for region, height, physical activity, Townsend deprivation index, education, employment, smoking, alcohol consumption measured at recruitment, ethnicity, diabetes status, non-steroidal anti-inflammatory drug use, energy intake, BMI, processed and red meat intake, total vegetable fruit intake, and female specific covariates: menopausal hormone therapy use and menopausal status.  g/day are mean and standard deviation within each quartile  P-values represent the p-values for wholegrains and refined grains modelled as 50 g/day increase in the model.  Abbreviations: CI, confidence intervals; g/day, grams per day; HR, hazard ratio, N, number of subjects; Q, quartile; ref, reference group. | | | |

| **Supplementary Table 6**. Sequential adjustment hazard ratios and 95% confidence intervals for intake of fibre sources with colorectal cancer risk (n=114,217) | | | | | | | | |
| --- | --- | --- | --- | --- | --- | --- | --- | --- |
| **Fibre and fibre sources** | **Q1** | **Q2** | **Q3** | **Q4** | **Per 5 gram increment** | ***χ*^2^** | ***χ*^2^ – % change** | ***P-trend*** |
| **Total fibre** |  |  |  |  |  |  |  |  |
| Minimally-adjusted* | 1 (ref) | 1.11 (0.94 - 1.30) | 1.01 (0.86 - 1.19) | 0.99 (0.84 - 1.17) | 0.97 (0.92 - 1.02) | 1.37 |  | 0.24 |
| Multivariable-adjusted** | 1 (ref) | 1.07 (0.90 - 1.27) | 0.96 (0.81 - 1.15) | 0.92 (0.76 - 1.11) | **0.94 (0.89 - 1.00)** | 3.98 | +189.3% | 0.05 |
| + BMI | 1 (ref) | 1.08 (0.91 - 1.27) | 0.98 (0.82 - 1.17) | 0.95 (0.78 - 1.14) | 0.95 (0.89 - 1.01) | 2.81 | +104.2% | 0.09 |
| + Processed/red meat intake | 1 (ref) | 1.08 (0.91 - 1.28) | 0.99 (0.83 - 1.18) | 0.97 (0.80 - 1.17) | 0.96 (0.90 - 1.02) | 1.93 | +40.5% | 0.16 |
|  |  |  |  |  |  |  |  |  |
| **Fibre from fruits and vegetables** |  |  |  |  |  |  |  |  |
| Minimally-adjusted* | 1 (ref) | 0.95 (0.81 - 1.12) | 0.99 (0.84 - 1.16) | 0.91 (0.77 - 1.07) | 0.93 (0.85 - 1.01) | 3.03 |  | 0.08 |
| Multivariable-adjusted** | 1 (ref) | 0.97 (0.82 - 1.14) | 1.01 (0.85 - 1.19) | 0.92 (0.78 - 1.09) | 0.93 (0.85 - 1.02) | 2.59 | -14.6% | 0.11 |
| + BMI | 1 (ref) | 0.98 (0.83 - 1.15) | 1.02 (0.86 - 1.20) | 0.93 (0.79 - 1.11) | 0.94 (0.86 - 1.02) | 2.21 | -27.1% | 0.14 |
| + Processed/red meat intake | 1 (ref) | 0.98 (0.83 - 1.15) | 1.02 (0.87 - 1.20) | 0.94 (0.80 - 1.12) | 0.94 (0.86 - 1.03) | 1.74 | -42.8% | 0.19 |
|  |  |  |  |  |  |  |  |  |
| **Fibre from vegetables** |  |  |  |  |  |  |  |  |
| Minimally-adjusted* | 1 (ref) | 1.09 (0.92 - 1.28) | 1.03 (0.87 - 1.21) | 1.03 (0.87 - 1.21) | 0.97 (0.86 - 1.11) | 0.16 |  | 0.69 |
| Multivariable-adjusted** | 1 (ref) | 1.09 (0.92 - 1.28) | 1.02 (0.87 - 1.21) | 1.01 (0.85 - 1.19) | 0.96 (0.84 - 1.09) | 0.45 | +186.4% | 0.50 |
| + BMI | 1 (ref) | 1.09 (0.93 - 1.29) | 1.03 (0.88 - 1.22) | 1.02 (0.86 - 1.20) | 0.96 (0.84 - 1.10) | 0.35 | +121.8% | 0.55 |
| + Processed/red meat intake | 1 (ref) | 1.09 (0.93 - 1.29) | 1.04 (0.88 - 1.22) | 1.02 (0.86 - 1.21) | 0.97 (0.85 - 1.11) | 0.21 | +32.8% | 0.65 |
|  |  |  |  |  |  |  |  |  |
| **Fibre from fruits** |  |  |  |  |  |  |  |  |
| Minimally-adjusted* | 1 (ref) | 0.96 (0.82 - 1.13) | **0.85 (0.72 - 1.00)** | **0.83 (0.70 - 0.97)** | **0.84 (0.73 - 0.97)** | 5.99 |  | 0.01 |
| Multivariable-adjusted** | 1 (ref) | 0.99 (0.85 - 1.16) | 0.89 (0.75 - 1.05) | 0.87 (0.73 - 1.03) | **0.87 (0.75 - 1.00)** | 3.70 | -38.3% | 0.05 |
| + BMI | 1 (ref) | 1.00 (0.85 - 1.17) | 0.90 (0.76 - 1.06) | 0.88 (0.74 - 1.04) | 0.88 (0.76 - 1.01) | 3.27 | -45.4% | 0.07 |
| + Processed/red meat intake | 1 (ref) | 1.00 (0.85 - 1.18) | 0.90 (0.76 - 1.06) | 0.89 (0.75 - 1.05) | 0.89 (0.77 - 1.02) | 2.79 | -53.5% | 0.10 |
|  |  |  |  |  |  |  |  |  |
|  |  |  |  |  |  |  |  |  |
|  |  |  |  |  |  |  |  |  |
|  |  |  |  |  |  |  |  |  |
|  |  |  |  |  |  |  |  |  |
|  | **Q1** | **Q2** | **Q3** | **Q4** | **Per 5 gram increase** | ***χ*^2^** | ***χ*^2^ – % reduction** | ***P-trend*** |
| **Fibre from wholegrains** |  |  |  |  |  |  |  |  |
| Minimally-adjusted* | 1 (ref) | 0.87 (0.74 - 1.02) | 0.90 (0.77 - 1.06) | **0.77 (0.65 - 0.90)** | **0.87 (0.79 - 0.96)** | 7.82 |  | 0.005 |
| Multivariable-adjusted** | 1 (ref) | 0.88 (0.75 - 1.04) | 0.93 (0.79 - 1.08) | **0.78 (0.66 - 0.92)** | **0.88 (0.80 - 0.97)** | 6.53 | -16.5% | 0.011 |
| + BMI | 1 (ref) | 0.89 (0.76 - 1.05) | 0.94 (0.80 - 1.10) | **0.80 (0.68 - 0.95)** | **0.90 (0.81 - 0.99)** | 4.84 | -38.1% | 0.028 |
| + Processed/red meat & total vegetable and fruit intake | 1 (ref) | 0.89 (0.76 - 1.05) | 0.94 (0.81 - 1.11) | **0.81 (0.69 - 0.96)** | **0.90 (0.82 - 1.00)** | 3.96 | -49.4% | 0.047 |
| *Minimally-adjusted model stratified for sex, age at recruitment (<45, 45-49, 50-54, 55-59, 60-64, ≥65 years) and adjusted for region (North-West England, North-Eastern England, Yorkshire & the Humber, West Midlands, East Midlands, South-East England, South-West England, London, Wales, and Scotland).  **Multivariable adjusted model further adjusted for height, physical activity, Townsend deprivation index, education, employment, smoking, alcohol consumption measured at recruitment, ethnicity, diabetes status, non-steroidal anti-inflammatory drug use, energy intake, female specific covariates: menopausal hormone therapy use and menopausal status.  + BMI further adjusted for body mass index categories.  + processed/red meat & total vegetable fruit intake further adjusted for red and processed meat consumption reported at recruitment and fruit and vegetables intake measured by averaged 24-hour dietary assessments.  χ^2^ and p-trend represent improvement of fit obtained from likelihood ratio tests for including fibre source (modelled as a 5 gram/day increase) variable into the model.  χ^2^ - % change calculated from the percentage difference in χ^2^ value using the minimally adjusted χ^2^ value as the reference group.  Abbreviations: BMI, body mass index; Q, quartile; ref, reference group. | | | | | | | | |

| **Supplementary Table 7.** Multivariable hazard ratios and 95% confidence intervals for carbohydrate and fibre sources with colorectal cancer risk restricting to participants who completed **≥3 24-hour dietary assessments** (N= 69,223; n cases = 734). | | | | | | |
| --- | --- | --- | --- | --- | --- | --- |
| **Carbohydrates, sugars, & starch** | **Q1** | **Q2** | **Q3** | **Q4** | **Per 5% energy increment** | ***P-trend*** |
| Total carbohydrates | 1 (ref) | 0.89 (0.72 - 1.08) | 0.90 (0.73 - 1.11) | 0.88 (0.69 - 1.11) | 0.95 (0.89 - 1.01) | 0.079 |
| Total sugar | 1 (ref) | 0.82 (0.67 - 1.00) | 0.87 (0.71 - 1.07) | **0.67 (0.53 - 0.86)** | **0.90 (0.84 - 0.96)** | 0.002 |
| Free sugars | 1 (ref) | 1.12 (0.91 - 1.36) | 0.87 (0.70 - 1.08) | **0.78 (0.62 - 0.97)** | **0.90 (0.82 - 0.98)** | 0.01 |
| Non-free sugars | 1 (ref) | 1.00 (0.82 - 1.22) | 0.86 (0.69 - 1.07) | 0.87 (0.69 - 1.10) | **0.92 (0.85 - 1.00)** | 0.04 |
| Total starch | 1 (ref) | 0.98 (0.80 - 1.21) | 1.03 (0.84 - 1.27) | 1.14 (0.92 - 1.42) | 1.04 (0.97 - 1.13) | 0.26 |
| Wholegrain starch | 1 (ref) | 0.90 (0.73 - 1.10) | 0.93 (0.76 - 1.14) | 0.84 (0.68 - 1.04) | 0.93 (0.84 - 1.03) | 0.15 |
| Refined grain starch | 1 (ref) | 0.96 (0.78 - 1.18) | 1.04 (0.84 - 1.28) | 1.22 (0.99 - 1.51) | 1.07 (1.00 - 1.14) | 0.070 |
| **Fibre** | **Q1** | **Q2** | **Q3** | **Q4** | **Per 5 g/day increment** | ***P-trend*** |
| Total fibre | 1 (ref) | 1.08 (0.87 - 1.33) | 0.94 (0.75 - 1.17) | 0.89 (0.70 - 1.14) | 0.96 (0.88 - 1.04) | 0.33 |
| Fibre from fruits and vegetables | 1 (ref) | 1.02 (0.83 - 1.25) | 0.91 (0.74 - 1.13) | 0.93 (0.75 - 1.15) | 0.94 (0.83 - 1.05) | 0.26 |
| Fibre from vegetables | 1 (ref) | 1.10 (0.90 - 1.35) | 0.91 (0.74 - 1.13) | 0.91 (0.74 - 1.14) | 0.89 (0.74 - 1.07) | 0.20 |
| Fibre from fruits | 1 (ref) | 1.07 (0.87 - 1.31) | 1.01 (0.82 - 1.25) | 0.92 (0.74 - 1.15) | 0.95 (0.79 - 1.15) | 0.63 |
| Fibre from wholegrains | 1 (ref) | 0.94 (0.76 - 1.15) | 0.92 (0.75 - 1.13) | 0.85 (0.69 - 1.06) | 0.89 (0.78 - 1.02) | 0.09 |
| Models stratified for sex and age at recruitment, and further adjusted for region, height, physical activity, Townsend deprivation index, education, employment, smoking, alcohol consumption measured at recruitment, ethnicity, diabetes status, non-steroidal anti-inflammatory drug use, energy intake, BMI, processed and red meat intake, total vegetable fruit intake (except for total fibre, fibre from vegetables and/or fruit, or non-free sugars is the exposure), and female specific covariates: menopausal hormone therapy use and menopausal status.  P-trend values represent the p-values for carbohydrate types/source modelled as 5% energy increase per day in the model and fibre by 5 g/day increase in the model.  Abbreviations: BMI, body mass index; g/day, grams per day; Q, quartile; ref, reference group. | | | | | | |

| **Supplementary Table 8.** Multivariable hazard ratios and 95% confidence intervals for carbohydrate and fibre sources with colorectal cancer risk **removing participants with <2 years of follow-up** (N=111,724; n cases = 978). | | | | | | |
| --- | --- | --- | --- | --- | --- | --- |
| **Carbohydrates, sugars, & starch** | **Q1** | **Q2** | **Q3** | **Q4** | **Per 5% energy increment** | ***P-trend*** |
| Total carbohydrates | 1 (ref) | 0.89 (0.75 - 1.06) | 0.93 (0.78 - 1.12) | 0.83 (0.67 - 1.01) | **0.94 (0.90 - 0.99)** | 0.02 |
| Total sugar | 1 (ref) | 0.84 (0.70 - 1.00) | **0.83 (0.69 - 0.99)** | **0.72 (0.59 - 0.89)** | **0.94 (0.88 - 0.99)** | 0.02 |
| Free sugars | 1 (ref) | 1.08 (0.91 - 1.29) | 0.94 (0.78 - 1.13) | 0.87 (0.72 - 1.06) | 0.96 (0.90 - 1.03) | 0.28 |
| Non-free sugars | 1 (ref) | 0.87 (0.73 - 1.04) | **0.79 (0.66 - 0.95)** | **0.78 (0.64 - 0.95)** | **0.91 (0.85 - 0.98)** | 0.008 |
| Total starch | 1 (ref) | 0.90 (0.75 - 1.07) | 0.96 (0.81 - 1.15) | 0.94 (0.78 - 1.14) | 0.99 (0.93 - 1.05) | 0.64 |
| Wholegrain starch | 1 (ref) | 0.90 (0.75 - 1.07) | 0.91 (0.77 - 1.09) | 0.84 (0.70 - 1.01) | 0.94 (0.86 - 1.02) | 0.12 |
| Refined grain starch | 1 (ref) | 0.91 (0.76 - 1.09) | 1.02 (0.85 - 1.21) | 1.04 (0.86 - 1.25) | 1.01 (0.96 - 1.07) | 0.68 |
| **Fibre** | **Q1** | **Q2** | **Q3** | **Q4** | **Per 5 g/day increment** | ***P-trend*** |
| Total fibre | 1 (ref) | 1.07 (0.89 - 1.29) | 1.00 (0.82 - 1.21) | 0.95 (0.77 - 1.17) | 0.95 (0.89 - 1.02) | 0.13 |
| Fibre from fruits and vegetables | 1 (ref) | 0.95 (0.79 - 1.14) | 1.02 (0.85 - 1.22) | 0.92 (0.76 - 1.11) | 0.93 (0.84 - 1.02) | 0.13 |
| Fibre from vegetables | 1 (ref) | 1.10 (0.91 - 1.31) | 1.04 (0.87 - 1.25) | 1.01 (0.84 - 1.22) | 0.94 (0.82 - 1.09) | 0.44 |
| Fibre from fruits | 1 (ref) | 0.94 (0.79 - 1.12) | 0.86 (0.72 - 1.04) | 0.83 (0.69 - 1.00) | 0.88 (0.75 - 1.03) | 0.10 |
| Fibre from wholegrains | 1 (ref) | 0.90 (0.75 - 1.08) | 0.97 (0.81 - 1.15) | **0.78 (0.65 - 0.94)** | **0.89 (0.80 - 1.00)** | 0.04 |
| Models stratified for sex and age at recruitment, and further adjusted for region, height, physical activity, Townsend deprivation index, education, employment, smoking, alcohol consumption measured at recruitment, ethnicity, diabetes status, non-steroidal anti-inflammatory drug use, energy intake, BMI, processed and red meat intake, total vegetable fruit intake (except for total fibre, fibre from vegetables and/or fruit, or non-free sugar), and female specific covariates: menopausal hormone therapy use and menopausal status.  P-trend values represent the carbohydrate types/source modelled as 5% energy increase per day in the model and fibre by 5 g/day increase in the model.  Abbreviations: BMI, body mass index; g/day, grams per day; Q, quartile; ref, reference group. | | | | | | |

# **Supplementary Figures**

Number of 24-hour dietary assessments completed by participants:

- Two: 44,994
- Three: 38,480
- Four: 26,032
- Five: 4,711

Recruitment

Oxford WebQ 24-hour dietary assessment

Second (n=75,549)

First

(n=31,832)

Fourth (n=82,514)

Fifth

(n=76,645)

Third (n=66,571)

WebQ subsample: completed a minimum of two WebQs (n=114,217)

2012

2006 -2009

2011

2010

End of recruitment

## **Supplementary Figure 1**. Dietary assessment for participants in the UK Biobank over time.

Numbers exclude participants who withdrew their consent, were diagnosed with a prevalent cancer before recruitment or before their last 24-hour dietary assessment, the 24-hour dietary assessment was deemed invalid due to reporting extreme energy intake or participant said they were ill or fasting on the respective day, did not complete a minimum of two 24-hour dietary assessments, or were censored before they completed their final 24-hour dietary assessment (please see supplementary figure 2 for further details).

## **
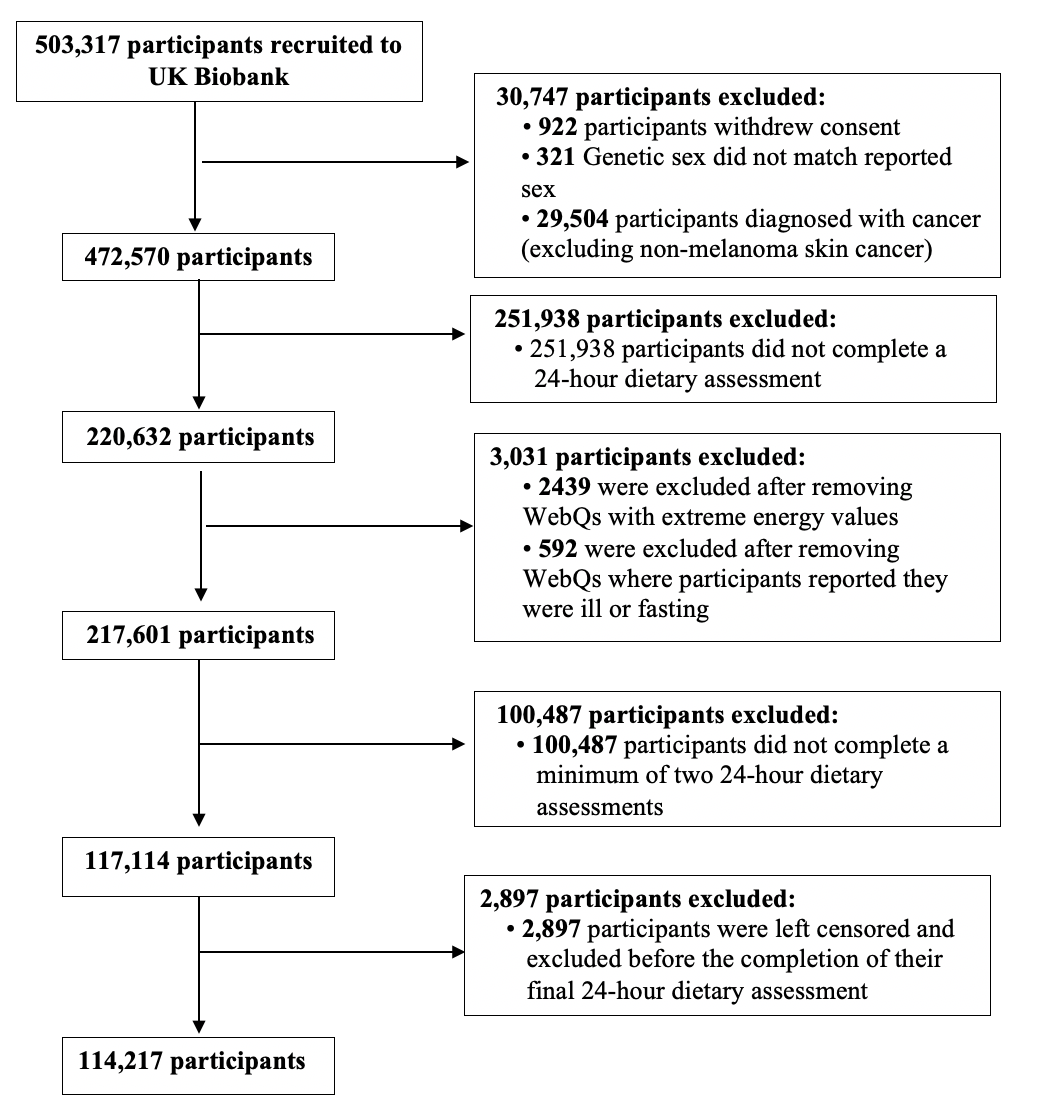
**

## **Supplementary Figure 2.** Flow chart of exclusion criteria for subsample of participants from the UK Biobank included in the main analyses.

## **Supplementary Figure 3.** Top food group contributors to types and sources of carbohydrates for participants in the 24-hour dietary assessment subsample (n=114,217).

Abbreviation: g/day, grams per day

## **Supplementary Figure 4.** Multivariable hazard ratios and 95% confidence intervals for carbohydrate and fibre sources with colorectal cancer risk **stratified by sex.**

Models stratified for age at recruitment, and further adjusted for region, height, physical activity, Townsend deprivation index, education, employment, smoking, alcohol consumption measured at recruitment, ethnicity, diabetes status, non-steroidal anti-inflammatory drug use, energy intake, BMI, processed and red meat intake, total vegetable fruit intake (except for total fibre, fibre from vegetables and/or fruit, or non-free sugars), and female specific covariates: menopausal hormone therapy use and menopausal status.

χ^2^ and p-value for heterogeneity represents improvement of fit obtained from likelihood ratio tests for including an interaction term between sex and carbohydrate type/source (modelled as a 5% energy increase) or fibre source (modelled as a 5 gram/day increase) into the model.

Abbreviations: CI, confidence interval; g/day, grams per day; HR, hazard ratio, N, number of participants.

## **Supplementary Figure 5.** Multivariable hazard ratios and 95% confidence intervals for carbohydrate and fibre sources with colorectal cancer risk **stratified by body mass index (<27 kg/m^2^ vs. ≥27 kg/m^2^).**

Models stratified for sex and age at recruitment, and further adjusted for region, height, physical activity, Townsend deprivation index, education, employment, smoking, alcohol consumption measured at recruitment, ethnicity, diabetes status, non-steroidal anti-inflammatory drug use, energy intake, processed and red meat intake, total vegetable fruit intake (except for total fibre, fibre from vegetables and/or fruit, or non-free sugars), and female specific covariates: menopausal hormone therapy use and menopausal status.

χ^2^ and p-value for heterogeneity represent improvement of fit obtained from likelihood ratio tests for including an interaction term between BMI subgroups and carbohydrate type/source (modelled as a 5% energy increase) or fibre source (modelled as a 5 gram/day increase) into the model.

Abbreviations: CI, confidence interval; HR, hazard ratio, N, number of participants.

## **Supplementary Figure 6.** Multivariable hazard ratios and 95% confidence intervals for carbohydrate and fibre sources with colorectal cancer risk **separated by tumour site (colon and rectal).**

Models stratified for sex and age at recruitment, and further adjusted for region, height, physical activity, Townsend deprivation index, education, employment, smoking, alcohol consumption measured at recruitment, ethnicity, diabetes status, non-steroidal anti-inflammatory drug use, energy intake, body mass index, processed and red meat intake, total vegetable fruit intake (except for total fibre, fibre from vegetables and/or fruit, or non-free sugars), and female specific covariates: menopausal hormone therapy use and menopausal status.

χ^2^ and p-value for heterogeneity represent Wald’s test for heterogeneity between tumour subgroups and carbohydrate types/sources (per 5% energy increase) or fibre source (per 5 g/day increase).

Abbreviations: CI, confidence interval; HR, hazard ratio, ICD-10, International Classification of Diseases Version 10.
